# Machine Learning-Based Screening and Experimental Validation of PIK3C2A as a Candidate Angiogenesis-Related Molecule in Pulmonary Hypertension

**DOI:** 10.3390/ijms27219421

**Published:** 2026-10-22

**Authors:** Yuna Wang, Di Zhao, Huangjin Xiao, Leyin Gao, Liwen Huang, Dan Bai, Deming Gou, Shuya Zhang

**Affiliations:** 1 Key Laboratory of Fertility Preservation and Maintenance of Ministry of Education, School of Basic Medical Sciences, Ningxia Medical University, Yinchuan 750004, China; wynmaggie@163.com (Y.W.);; 2 Ningxia Basic Medical Research Center, School of Basic Medical Sciences, Ningxia Medical University, Yinchuan 750004, China; 3 College of Life Sciences and Oceanography, Shenzhen University, Shenzhen 518060, China

**Keywords:** PH, angiogenesis, PIK3C2A, machine learning, single-cell RNA sequencing, biomarker

## Abstract

Pulmonary hypertension (PH) is a progressive cardiopulmonary disease characterized by pulmonary vascular remodeling and dysregulated angiogenesis. However, the angiogenesis-related molecular mechanisms underlying PH remain incompletely understood, and reliable diagnostic biomarkers are still lacking. Therefore, To identify angiogenesis-related key molecules in PH through integrated transcriptomic analyses and experimental validation, we aimed to establish a candidate molecular signature and evaluate the discriminative potential of *PIK3C2A*. Specifically, GSE15197 was used as the primary bulk-transcriptomic discovery dataset, whereas GSE1909 for cross-species validation, and human pulmonary artery single-cell RNA sequencing data (GSE210248) for cellular characterization. Moreover, Functional enrichment, Weighted Gene Co-expression Network Analysis (WGCNA), immune infiltration analysis, and three machine learning algorithms—logistic regression, random forest (RF), and Least Absolute Shrinkage and Selection Operator (LASSO) were performed to identify model genes. In addition, Single-cell analyses were performed using GSE210248, followed by quantitative real-time polymerase chain reaction (qRT-PCR) validation of candidate-gene expression in monocrotaline (MCT) and Sugen5416 plus hypoxia (SuHx) rat models. As a result, Forty-three angiogenesis-related differentially expressed genes (ARDEGs) were identified and were enriched in terms and pathways related to angiogenesis, vascular development, extracellular matrix organization, and HIF-1 signaling. Four model genes (*DCN*, *DKK1*, *PIK3C2A*, and *UNC5B*) were identified and showed moderate-to-high discrimination between PH and control samples in GSE15197. Among these genes, *PIK3C2A* showed its highest expression in endothelial cells, while complementary immune-infiltration and CellChat (version 2.1.1) analyses characterized the broader immune–stromal context of the angiogenesis-related signature. qRT-PCR further confirmed significant downregulation of *PIK3C2A* in the lungs and pulmonary arteries of MCTand SuHxinduced PH models (*p* < 0.05). In conclusion, An angiogenesis-related candidate molecular signature comprising *DCN*, *DKK1*, *PIK3C2A* and *UNC5B* was identified in PH. Among these genes, *PIK3C2A* showed preferential endothelial expression and was downregulated in both the MCT and SuHx rat PH models, supporting its further evaluation as a candidate molecule associated with PH.

## 1. Introduction

Pulmonary hypertension (PH) represents a critical cardiopulmonary disorder defined by heightened pulmonary arterial pressure and escalated pulmonary vascular resistance. PH encompasses multiple etiological and clinical subgroups, including pulmonary arterial hypertension (PAH), which is a distinct subtype characterized by precapillary hemodynamics and specific pulmonary vascular remodeling. The epidemiological profile of PH fluctuates significantly depending on specific subtypes and comorbidities, while disease progression invariably leads to profound functional impairment and right ventricular failure [1]. The etiology of PH is multifaceted, arising from the convergence of diverse mechanisms such as endothelial dysfunction [2], aberrant smooth muscle cell proliferation [3], inflammatory cascades [2], and metabolic reprogramming [4]. The 2022 ESC/ERS guidelines emphasize a stepwise diagnostic pathway from clinical suspicion to echocardiographic detection and confirmation by right heart catheterization [5]. Although right heart catheterization remains the diagnostic reference standard, it is invasive and provides limited information regarding molecular disease phenotypes and the biological processes underlying vascular remodeling. Identification of molecular signatures that reflect PH pathobiology may therefore help to prioritize candidate biomarkers and biologically relevant pathways for further investigation.

Angiogenesis and vascular repair are integral components of pulmonary vascular homeostasis, making their dysregulation relevant to pulmonary vascular remodeling in PH. At specific disease stages and within particular pulmonary vascular compartments, impaired angiogenic repair may contribute to microvascular rarefaction, worsening hypoxia, and PH progression [6]. More broadly, angiogenesis-related remodeling can involve dysregulated vascular repair, endothelial activation, immune-cell recruitment, and remodeling of vascular niches [7,8,9]. Angiogenesis-related genes (ARGs) have been linked to endothelial function [10] and vascular remodeling [11]. However, the specific ARGs and regulatory networks associated with PH and pulmonary vascular remodeling remain incompletely characterized. Among angiogenesis-related regulators, phosphatidylinositol-4-phosphate 3-kinase catalytic subunit type 2 alpha (*PIK3C2A*) has established roles in endothelial integrity and angiogenic function. *PIK3C2A* contributes to endothelial barrier function partly through regulation of VE-cadherin trafficking to adherens junctions [12]. Notably, dysfunction or downregulation of *PIK3C2A* has been associated with increased vascular permeability and impaired angiogenic function [13], features that are relevant to pulmonary vascular remodeling. However, whether *PIK3C2A* expression is altered in the pulmonary vasculature of PH and whether its expression changes consistently across distinct pathogenic contexts remain insufficiently characterized.

To address this gap, we integrated human bulk transcriptomic analysis, cross-species expression validation, pulmonary-artery single-cell RNA sequencing (scRNA-seq), and complementary rat PH models to prioritize angiogenesis-related candidates across biological scales and pathogenic contexts. We first identified angiogenesis-related differentially expressed genes (ARDEGs) in human PH lung tissue and characterized their functional enrichment. We then combined Weighted Gene Co-expression Network Analysis (WGCNA), angiogenesis-score stratification, immune-infiltration analysis, and three machine-learning approaches to prioritize candidate genes. Human pulmonary-artery scRNA-seq data were then used to characterize candidate-gene localization and to explore intercellular communication and pseudotime-associated expression dynamics. Finally, candidate gene expression was validated in rat PH models. The overall objective of this study was to define angiogenesis-related molecular alterations in PH, prioritize candidate genes with discriminative potential through integrative analysis, and experimentally assess the most promising candidate across complementary rat PH models.

## 2. Results

### 2.1. Identification of Angiogenesis-Related Differentially Expressed Genes in PH

**Figure 1:**
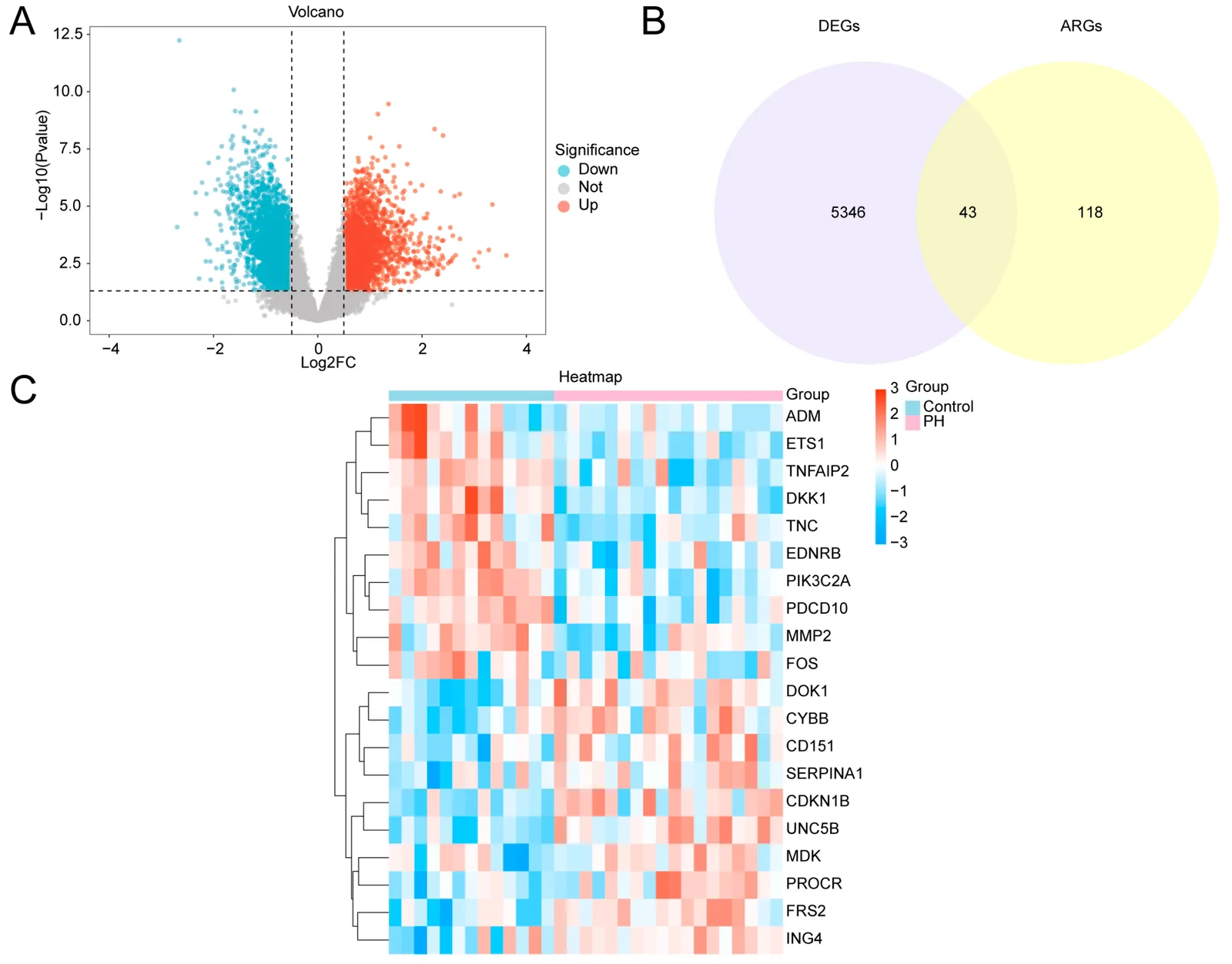
Identification of angiogenesis-related differentially expressed genes (ARDEGs) in PH. (**A**). Volcano plot of genes differentially expressed between PH and control samples. Genes were considered differentially expressed when ∣log2FC∣ > 0.5 and *p* < 0.05. (**B**). Venn diagram of differentially expressed genes (DEGs) and ARGs. (**C**). Heat map of ARDEGs.

Following normalization, GSE15197 expression distributions were comparable across samples. Differential-expression analysis identified 5389 PH-associated candidate DEGs meeting the predefined nominal significance and effect size thresholds. Among these 5389 genes, 4565 also met the Benjamini–Hochberg-adjusted significance criterion of adjusted *p* < 0.05 under the same effect-size threshold. There were 2652 up-regulated genes (log2FC > 0.5 and nominal *p* value < 0.05) and 2737 down-regulated genes (log2FC < −0.5 and nominal *p* value < 0.05) (Figure 1A). Intersecting the GSE15197 DEGs with the subset of 161 ARGs that were comparable across species retained after gene-set filtering yielded 43 candidate ARDEGs (Figure 1B). The gene list is shown in Table S2. Because the ARDEG set was derived using the prespecified exploratory nominal *p*-value threshold, these 43 genes are reported as candidate ARDEGs rather than as uniformly FDR-controlled findings. Heatmap visualization showed distinct expression patterns of representative ARDEGs between PH and control samples. Representative ARDEGs included *ADM*, *ETS1*, *TNFAIP2*, *DKK1*, *TNC*, *EDNRB*, *PIK3C2A*, *PDCD10*, *MMP2*, *FOS*, *DOK1*, *CYBB*, *CD151*, *SERPINA1*, *CDKN1B*, *UNC5B*, *MDK*, *PROCR*, *FRS2* and *ING4* (Figure 1C).

### 2.2. ARDEGs Are Enriched in Vascular Development, Extracellular Matrix, Focal Adhesion, and PH-Relevant Signaling Pathways

Gene Ontology (GO) enrichment analysis of ARDEGs revealed that the enriched biological processes were mainly related to the regulation of angiogenesis and vascular development, including regulation of angiogenesis, regulation of vasculature development, and their positive regulatory processes. In addition, ARDEGs were significantly enriched in processes associated with cellular responses to peptide hormones. At the cellular component level, these genes were predominantly enriched in a collagen-containing extracellular matrix, cell–substrate junction, focal adhesion, secretory granule lumen, and cytoplasmic vesicle lumen. Molecular function analysis further suggested that ARDEGs may be involved in several molecular functions, including DNA-binding transcription activator activity, RNA polymerase II-specific DNA-binding transcription factor binding, integrin binding, and transcription coregulator binding (Figure S3A).

Kyoto Encyclopedia of Genes and Genomes (KEGG) pathway analysis showed that ARDEGs were significantly enriched in pathways including proteoglycans in cancer, AGE-RAGE signaling pathway in diabetic complications, lipid and atherosclerosis, microRNAs in cancer, and HIF-1 signaling pathway. Collectively, these enrichment analyses suggested that ARDEGs may participate in extracellular matrix remodeling, regulation of cell adhesion, hypoxia-associated signaling pathways, inflammatory and metabolic responses, and transcriptional regulation (Figure S3B–E; Table 1).

### 2.3. Construction of Pulmonary Hypertension Co-Expression Modules via Weighted Gene Co-Expression Network Analysis and Screening of Angiogenesis-Related Differential Genes

**Figure 2:**
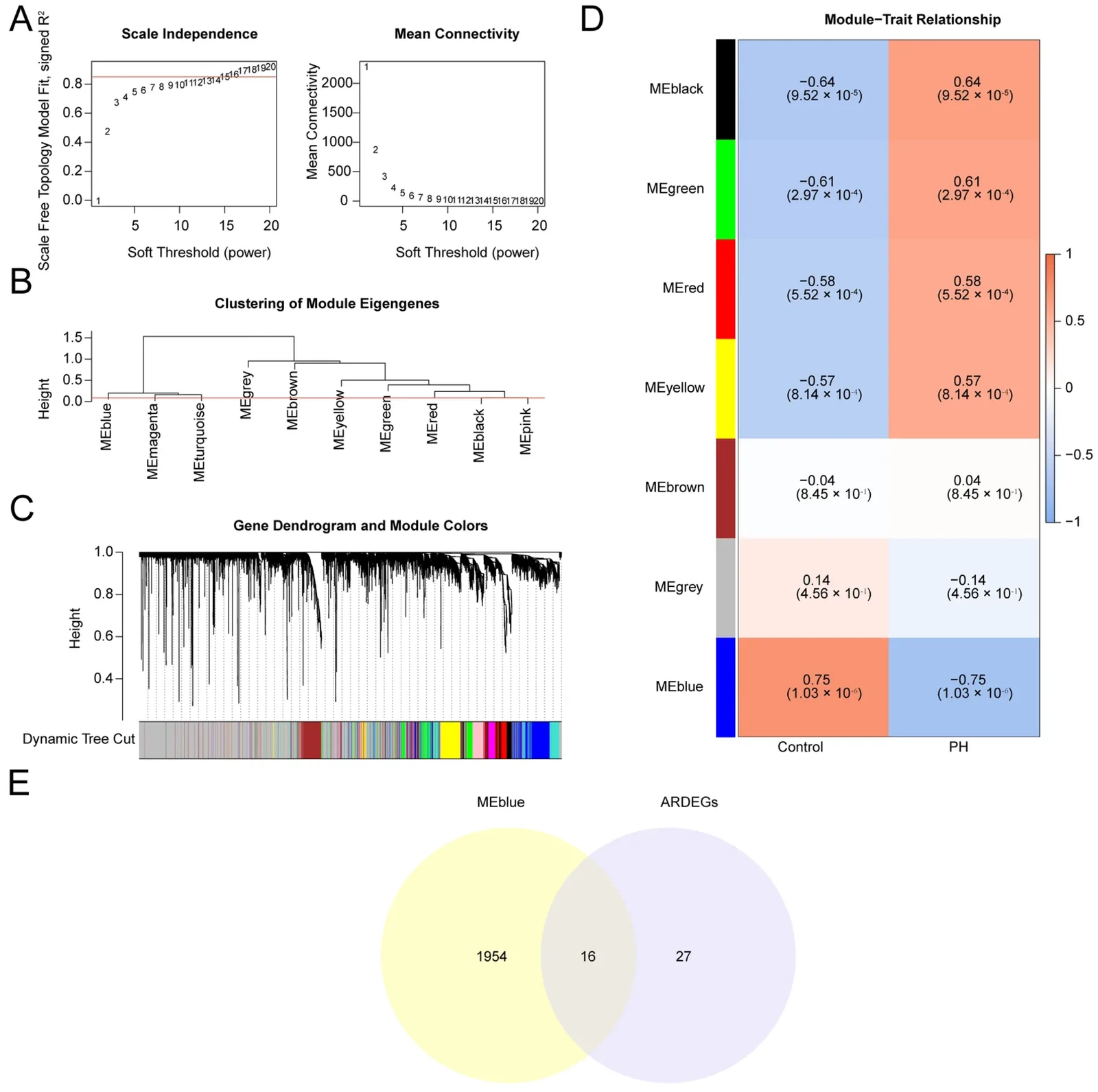
WGCNA analysis. (**A**). Characterization of the optimal soft-thresholding power, illustrating the scale-free topology fit of the WGCNA (left) and mean connectivity across varying threshold values (right). (**B**). Dendrogram depicting the hierarchical clustering of genes exhibiting the highest 50% variance. (**C**). Modular assignment of these high-variance genes, with the upper section displaying the clustering dendrogram and the lower section delineating distinct gene modules. The black dendrogram at the top shows hierarchical clustering of genes based on expression dissimilarity, with the vertical axis representing gene dissimilarity (height); genes with highly similar expression patterns merge at the same height. The colored bars below the dendrogram (Dynamic Tree Cut) represent different gene modules identified by WGCNA. Genes within the same colored block belong to the same module and share highly correlated expression patterns. These colors are automatically assigned by the WGCNA package and are used for subsequent module-based enrichment analysis. (**D**). Heatmap visualizing correlation coefficients between the identified gene modules and the PH versus control cohorts. (**E**). Venn diagram highlighting the intersection between 43 ARDEGs and those assigned to the MEblue module. Correlation strength is defined by the absolute coefficient (|r|): <0.3 indicates negligible association, 0.3–0.5 indicates weak association, 0.5–0.8 indicates moderate association, and >0.8 indicates strong association. Color coding denotes directionality, with red indicating positive correlations and blue indicating negative correlations.

To identify gene modules associated with angiogenesis-related traits, WGCNA was conducted using the highly variable genes (top 50%) from dataset GSE15197. The optimal soft-thresholding power was determined based on scale-free topology analysis and network connectivity evaluation (Figure 2A). A power value of 16 was selected, corresponding to a scale-free topology fit index of 0.85. Subsequently, the co-expression network was constructed, and genes with similar expression patterns were grouped through hierarchical clustering (Figure 2B). With the module-merging threshold set at 0.2, the genes exhibiting the top 50% of variance were clustered into seven co-expression modules. The relationships between individual genes and the merged modules were subsequently visualized (Figure 2C). Based on the expression patterns of the module genes, the associations between each module and the PH and control groups were then assessed (Figure 2D). Module–trait correlation analysis revealed that the MEblue module showed the strongest association with the phenotype of interest (|r| > 0.7, nominal *p* < 0.05) and was therefore prioritized as an exploratory candidate module for subsequent analyses. Module–phenotype associations were evaluated using nominal *p* values and were not subjected to unified multiple-testing or FDR correction; accordingly, MEblue was not interpreted as an FDR-controlled significant module. The MEblue module contained 1970 genes, which represented the complete set of genes assigned to this co-expression module. These genes were not considered individually significant genes or hub genes solely on the basis of their module membership. To identify candidate genes, the 43 ARDEGs were intersected with the 1970 genes assigned to the MEblue module. This intersection yielded 16 candidate genes: *ROCK1*, *MMP2, PDCD10*, *CYBB*, *DKK1*, *EDNRB*, *SGPP1*, *PIK3C2A*, *TNFAIP2*, *UNC5B*, *NFE2L2*, *NRAS*, *DCN*, *CD151*, *METAP2*, and *HIF1A*. The overlap was visualized using a Venn diagram (Figure 2E), as shown in Table S3.

### 2.4. The Angiogenesis Score Discriminates PH and Captures Pathway Remodeling

**Figure 3:**
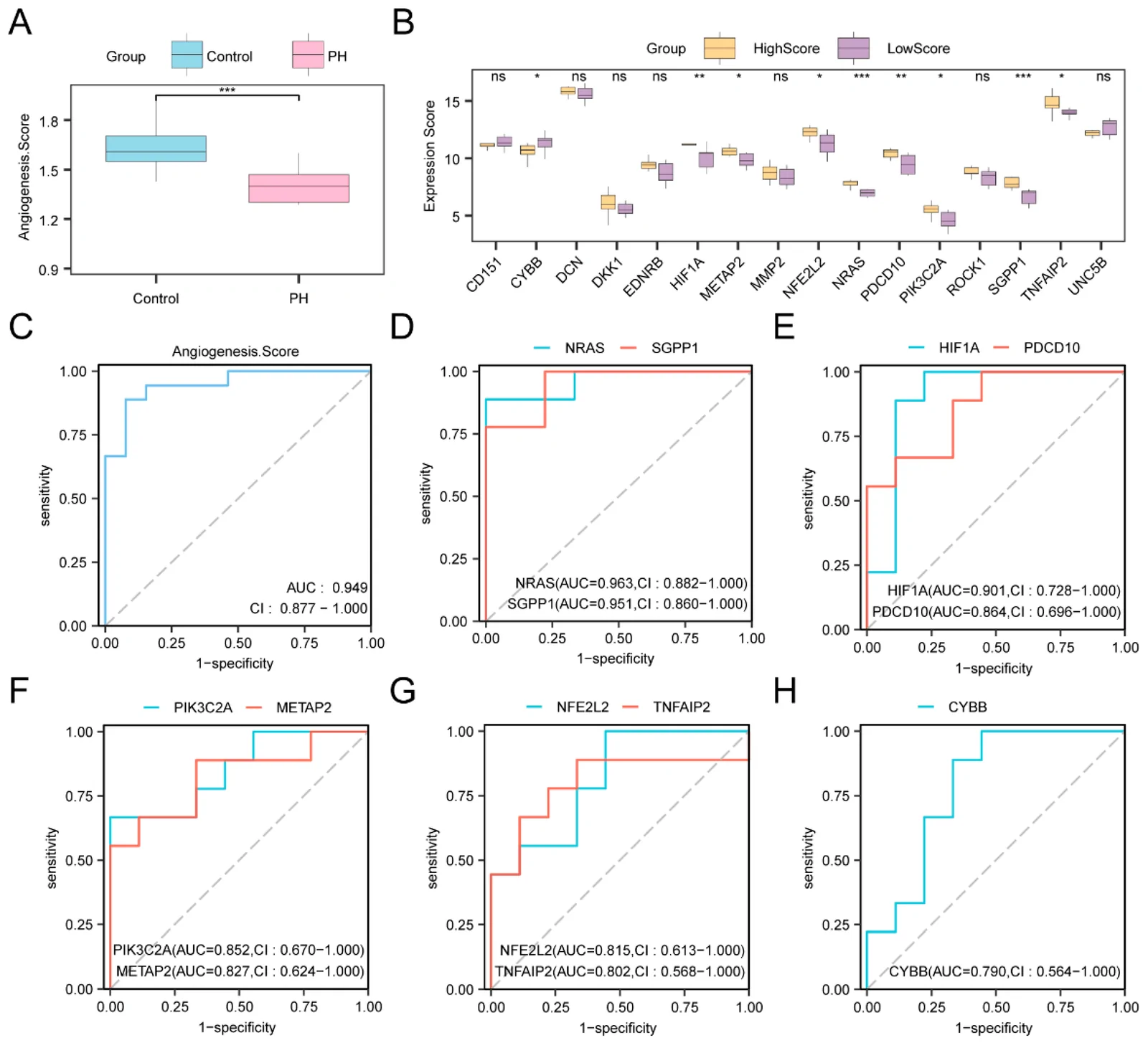
Construction of an angiogenesis-related score using single-sample gene set enrichment analysis (ssGSEA). (**A**). Group comparison of A. score between PH and control groups in dataset GSE15197. (**B**). Group comparison of module genes between the HighScore group and the LowScore group of pulmonary hypertension (PH) samples. (**C**). Receiver operating characteristic (ROC) curve of A. score in dataset GSE15197. The blue line represents the predictive performance of the A.score in distinguishing the disease group from the control group. D–H. ROC curves of module genes *NRAS* and *SGPP1* (**D**), *HIF1A* and *PDCD10* (**E**), *PIK3C2A* and *METAP2* (**F**), *NFE2L2* and *TNFAIP2* (**G**), *CYBB* (**H**) in PH samples. ns indicates *p* value ≥ 0.05, not statistically significant; *, *p* < 0.05; **, *p* < 0.01; ***, *p* < 0.001, with increasing asterisks reflecting a higher level of significance. The closer the AUC is to 1, the better the apparent discriminatory capacity; 0.7–0.9 indicates moderate apparent discriminatory capacity, and >0.9 indicates high discriminative performance. The dashed line indicates the "Reference Line", which corresponds to a model with no discriminatory ability.

The Angiogenesis Score (A.score) was significantly lower in PH samples than in controls (*p* < 0.001) and showed strong discrimination between PH and control samples (area under the curve [AUC] = 0.949, 95% CI = 0.877–1.000; Figure 3A–C). Among individual module genes, *NRAS* and *SGPP1* showed high discrimination between score-defined groups (AUC = 0.963 and 0.951, respectively), while *HIF1A*, *PDCD10*, *PIK3C2A*, *METAP2*, *NFE2L2*, *TNFAIP2* and *CYBB* also displayed moderate-to-high discriminative performance (Figure 3D–H).

GSVA of the c2.cp.v2023.2.Hs.symbols gene-set collection identified pathway-level differences between HighScore and LowScore PH samples in GSE15197, with detailed results provided in Table S4. GSVA comparing high-score and low-score PH samples revealed pathway-level differences involving antigen processing and presentation, PDZ-protein-related cell–cell junction signaling, PERK-ATF4 stress-response pathways, cytosolic tRNA aminoacylation, mitotic progression, diphthamide synthesis, propionyl-CoA catabolism, ketone-body metabolism, small-RNA silencing, lectin complement activation, PPARG signaling, fatty-acid cycling, and calcium transport (Figure S4A,B). These results suggest that angiogenesis-score stratification reflects a broader vascular–immune–metabolic remodeling state rather than isolated angiogenic activity.

### 2.5. Immune Infiltration Analysis of High- and Low-Score Groups (CIBERSORT)

CIBERSORT was used to estimate the relative proportions of 22 immune cell types in PH samples from the HighScore and LowScore groups (Figure S5A). In the LowScore group, M2 macrophages and resting mast cells exhibited the strongest positive correlation (r = 0.876, nominal *p* < 0.05). Correlation analysis between module genes and immune cell populations showed that EDNRB was positively correlated with resting memory CD4^+^ T cells (r = 0.881, nominal *p* < 0.05) but negatively correlated with M1 macrophages (r = −0.867, nominal *p* < 0.05) (Figure S5B,D). In the HighScore group, γδ T cells and resting dendritic cells displayed the strongest positive correlation (r = 1.000, nominal *p* < 0.05). Moreover, PIK3C2A was positively correlated with activated mast cells (r = 0.917, nominal *p* < 0.05) and negatively correlated with M2 macrophages (r = −0.967, nominal *p* < 0.05) (Figure S5C,E).

### 2.6. Machine Learning Prioritizes DCN, DKK1, PIK3C2A and UNC5B

**Figure 4:**
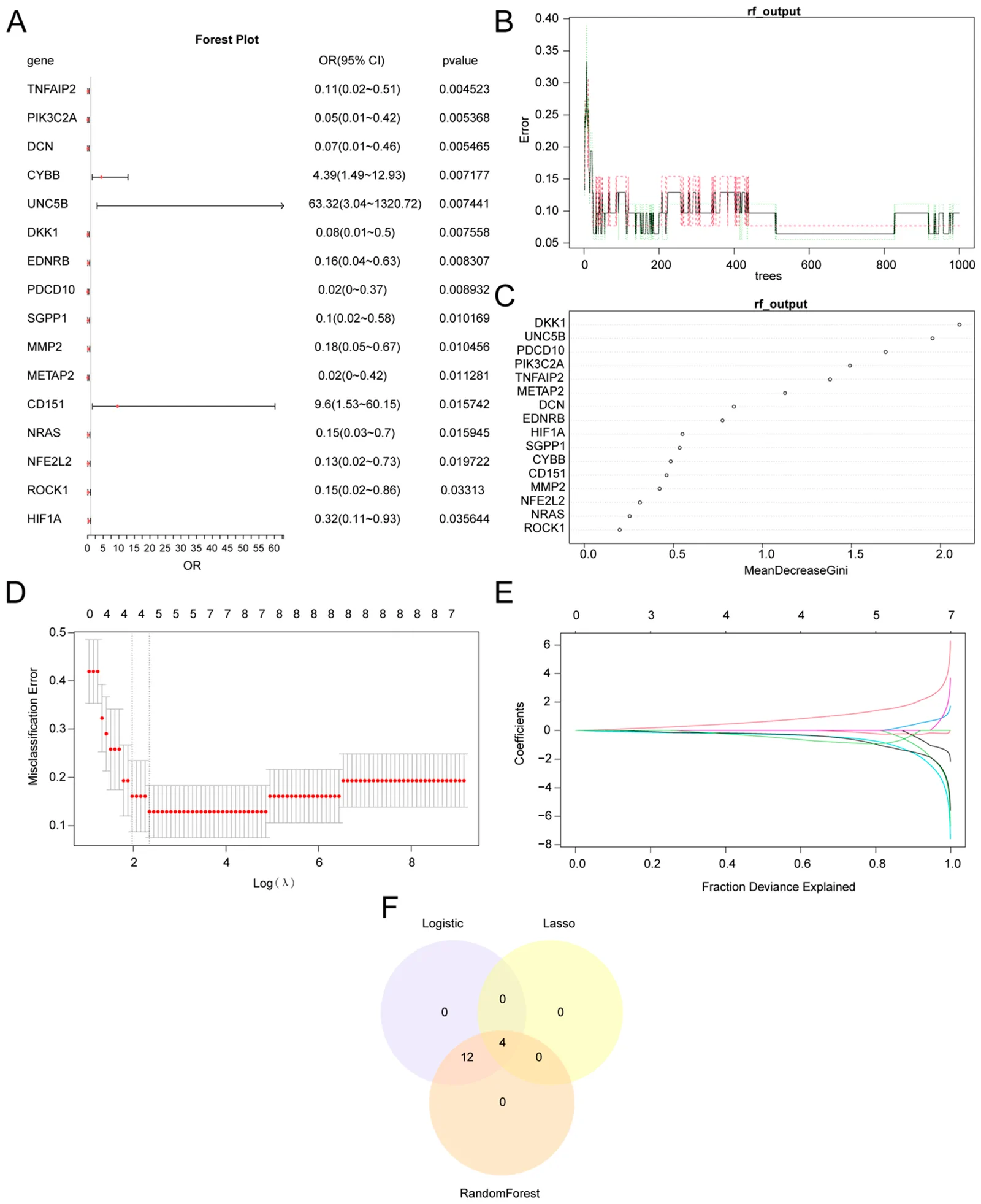
Machine-learning-based identification of model genes. (**A**). Forest plot of univariate logistic regression for the 16 module genes. Vertical dashed line: at X-axis tick 1, the null reference line (OR = 1). Horizontal line segment: represents the 95% confidence interval (95% CI) of the odds ratio (OR) for the corresponding gene. Black square: located at the midpoint of the line segment, representing the point estimate of the OR; the size of the square reflects the weight. X-axis: OR (odds ratio); OR > 1 indicates a risk factor, and OR < 1 indicates a protective factor. (**B**,**C**). Random forest (RF) algorithm. (**B**). RF error plot showing the overall out-of-bag (OOB) error rate and class-specific error rates versus the number of trees. X-axis (trees): number of decision trees constructed (0–1000). Y-axis (Error): OOB (out-of-bag) error. Black line: trend of the overall random forest model’s out-of-bag error as the number of trees changes. Colored lines (red/green): classification error for each of the two groups (case/control).Trend interpretation: as the number of trees increases, the error decreases rapidly and then plateaus, indicating that the model has converged. (**C**). Variable importance plot based on MeanDecreaseGini. (**D**,**E**). LASSO regression model. Y-axis: gene names, sorted from top to bottom by gene importance from high to low. X-axis (MeanDecreaseGini): mean decrease in Gini, used as the gene importance score. Black dots: each dot corresponds to one gene. (**D**). Cross-validation misclassification error curve as a function of log(λ), used to select the penalty parameter. X-axis (Log λ): log value of the penalty parameter λ. Y-axis (Misclassification Error): cross-validation misclassification error. Red dots: mean misclassification error corresponding to different λ values. Gray vertical error bars: standard error of the error. Two vertical dashed lines: the left dashed line islambda. min(the λ with the minimum cross-validation error); the right dashed line lambda.1se, the λ value corresponding tothe parsimonious model with fewer variables within 1 standard error of the minimum error. (**E**). LASSO coefficient profile plot. X-axis (Fraction Deviance Explained): the proportion of deviance explained by the model. Y-axis (Coefficients): the regression coefficients of the genes. Colored lines: each line represents the change in the regression coefficient of one gene. (**F**). Venn diagram showing the intersection of genes selected by multiple machine learning algorithms. Three colored circles: represent the gene sets obtained by three screening methods, respectively: purple for Logistic, yellow for Lasso, and orange for RandomForest.

Univariate logistic regression identified all 16 MEblue module genes as statistically associated with PH status (all *p* < 0.05; Figure 4A). Random forest (RF) analysis evaluated all 16 genes and ranked them according to their MeanDecreaseGini importance values (Figure 4B,C). Because the lowest average error rate in the repeated 10-fold cross-validation corresponded to all 16 input variables, RF did not further reduce the number of genes. Least Absolute Shrinkage and Selection Operator (LASSO) regression selected four genes at the minimum cross-validated error penalty parameter (lambda.min): *DKK1*, *PIK3C2A*, *UNC5B* and *DCN* (Figure 4D,E). Therefore, the reduction from the initial 16 genes to the final four-gene set was primarily achieved by LASSO regression. The four LASSO-selected genes all satisfied the univariate logistic regression significance criterion and were included in the RF importance rankings. Figure 4F illustrates the overlap among the gene sets evaluated by the three approaches: all 16 genes were included in the logistic regression and RF-related gene sets, whereas LASSO selected four genes. These four genes were therefore defined as the candidate model genes.

GeneMANIA generated a functional-association network comprising the four model genes and 20 predicted functionally related partners, with connections representing relationships such as co-expression and shared protein domains (Figure S6). Single-gene GSEA connected the model genes to apoptosis-related, inflammatory, pseudopodia/haptotaxis, stress-response, and heart-failure-associated gene sets (Figure S7; Table S5).

### 2.7. Immune Cell Infiltration Landscape in Pulmonary Hypertension and Its Correlation with Model Genes Based on ssGSEA

Using the GSE15197 dataset, the infiltration abundance of 28 immune cells was calculated via Single-Sample Gene-Set Enrichment Analysis (ssGSEA). As shown in Figure S8A, 13 immune-cell populations showed nominally significant differences between the groups; however, after Holm adjustment for multiple comparisons, only three populations remained significant (Holm-adjusted *p* < 0.05): central memory CD4 T cells, immature dendritic cells, and natural killer cells. The correlation heatmap (Figure S8B) indicated that most immune cell populations were strongly correlated with one another, with the strongest positive correlation observed between Gamma delta T cell and Regulatory T cells. The correlation bubble plot (Figure S8C) showed multiple associations between model genes and immune-cell signatures; PIK3C2A showed the strongest positive correlation with central memory CD4 T cells (r = 0.675, nominal *p* < 0.05), whereas DCN showed the strongest negative correlation with Type 17 T helper cells (r = −0.741, nominal *p* < 0.05). These findings indicate correlations between angiogenesis-related gene expression and inferred immune-cell signatures in PH.

### 2.8. Differential Expression Verification and ROC Analysis

**Figure 5:**
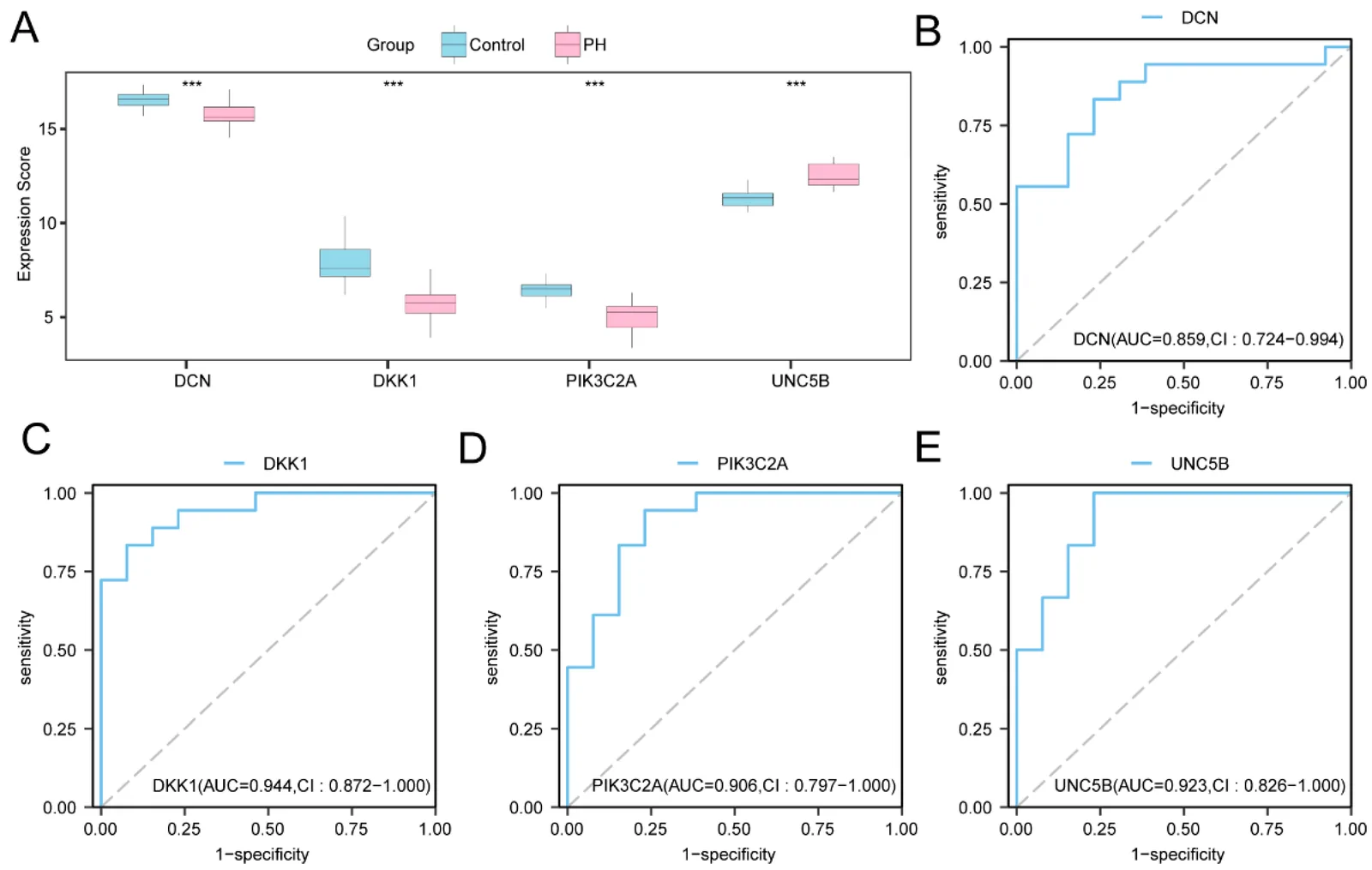
Model gene expression profiles and diagnostic ROC curves. (**A**). Comparison of model gene expression between the PH and control groups in the GSE15197 dataset. (**B**–**E**). ROC curves of model genes *DCN* (**B**), *DKK1* (**C**), *PIK3C2A* (**D**), and *UNC5B* (**E**) in dataset GSE15197. *** indicates a *p* value < 0.001, representing a highly significant difference. An AUC value closer to 1.0 indicates better apparent discriminatory capacity. An AUC value > 0.9 indicates high apparent discriminatory capacity, whereas an AUC value between 0.7 and 0.9 indicates moderate apparent discriminatory capacity within the corresponding cohort. The dashed line is the "Reference Line", representing the performance of a model with no discrimination ability.

In GSE15197, *DKK1*, *PIK3C2A*, *UNC5B* and *DCN* all showed significant expression differences between PH and control samples (all *p* < 0.001; Figure 5A). ROC analysis showed that the AUCs of *DCN*, *DKK1*, *PIK3C2A*, and *UNC5B* for discriminating patients with PH from controls were 0.859 (95% CI: 0.724–0.994), 0.944 (95% CI: 0.872–1.000), 0.906 (95% CI: 0.797–1.000), and 0.923 (95% CI: 0.826–1.000), respectively (Figure 5B–E).

In the cross-species validation dataset GSE1909, *UNC5B* and *DCN* differed significantly between the PH and comparison groups (*p* < 0.05 and *p* < 0.001, respectively; Figure S9A). ROC analysis showed that the AUCs of *DCN*, *DKK1*, *PIK3C2A*, and *UNC5B* for discriminating PH samples from controls were 0.909 (95% CI: 0.817–1.000), 0.491 (95% CI: 0.295–0.686), 0.480 (95% CI: 0.285–0.675), and 0.731 (95% CI: 0.565–0.898), respectively (Figure S9B–E).

### 2.9. Single-Cell RNA-Seq Localizes Model Gene Expression to Stromal and Vascular Compartments

**Figure 6:**
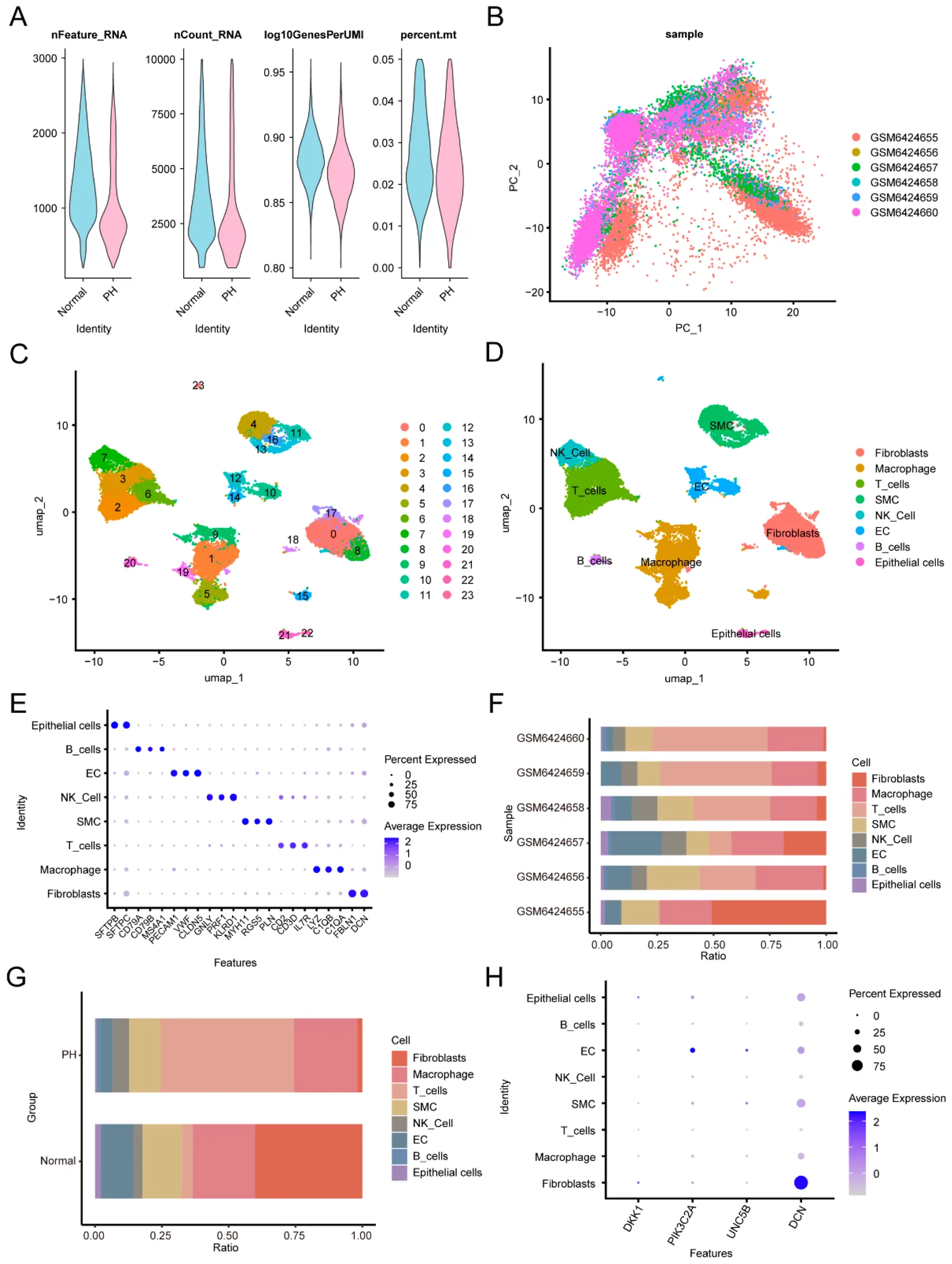
scRNA-seq analysis reveals cell-type-specific expression patterns of model genes in PH pulmonary artery tissue. (**A**). Violin plots depicting gene expression distributions within the GSE210248 cohort. (**B**). PCA illustrating transcriptional variance across distinct samples. (**C**). UMAP embedding of 21,730 cells, resolved into 24 distinct clusters. (**D**). Cell type annotation assigned to eight lineages—endothelial cells (EC), macrophages, natural killer (NK) cells, B cells, epithelial cells, fibroblasts, T cells, and smooth muscle cells (SMC)—using the R packages *SingleR* and marker gene profiles. (**E**). Bubble plot visualizing marker gene expression intensity across the eight identified cell populations. (**F**). Bar charts quantifying cellular composition ratios among individual samples. (**G**). Bar graphs comparing cell type proportions between experimental groups. (**H**). Bubble plot representation of expression metrics for the four model genes; color intensity correlates with expression magnitude, while circle size reflects the fraction of expressing cells. PCA, principal component analysis; UMAP, uniform manifold approximation and projection; UMI, unique molecular identifier.

After quality control, 21,730 cells from GSE210248 were analyzed. UMAP clustering identified 24 clusters that were annotated into eight major cell types: fibroblasts, macrophages, T cells, smooth muscle cells, natural killer cells, endothelial cells, B cells, and epithelial cells (Figure 6A–D). Canonical marker-gene expression supported the annotation, including *FBLN1* and *DCN* for fibroblasts, *LYZ/C1QA/C1QB* for macrophages, *CD2/CD3D/IL7R* for T cells, *MYH11/RGS5/PLN* for smooth muscle cells, *GNLY/PRF1/KLRD1* for natural killer cells, *CD79A/CD79B/MS4A1* for B cells, *PECAM1/VWF/CLDN5* for endothelial cells, and *SFTPB/SFTPC* for epithelial cells (Figure 6E). Cell-type proportions varied across individual samples and showed descriptive differences between PH and control groups (Figure 6F,G). Dot plots and UMAP feature plots demonstrated that *DCN* was predominantly expressed in fibroblasts and *PIK3C2A* showed the highest expression level in endothelial cells, while *DKK1* had relatively sparse expression in the scRNA-seq dataset (Figure 6H and Figure S10).

### 2.10. CellChat and Pseudotime Analyses Connect the Signature to Immune–Stromal Communication and Cellular-State Transitions

**Figure 7:**
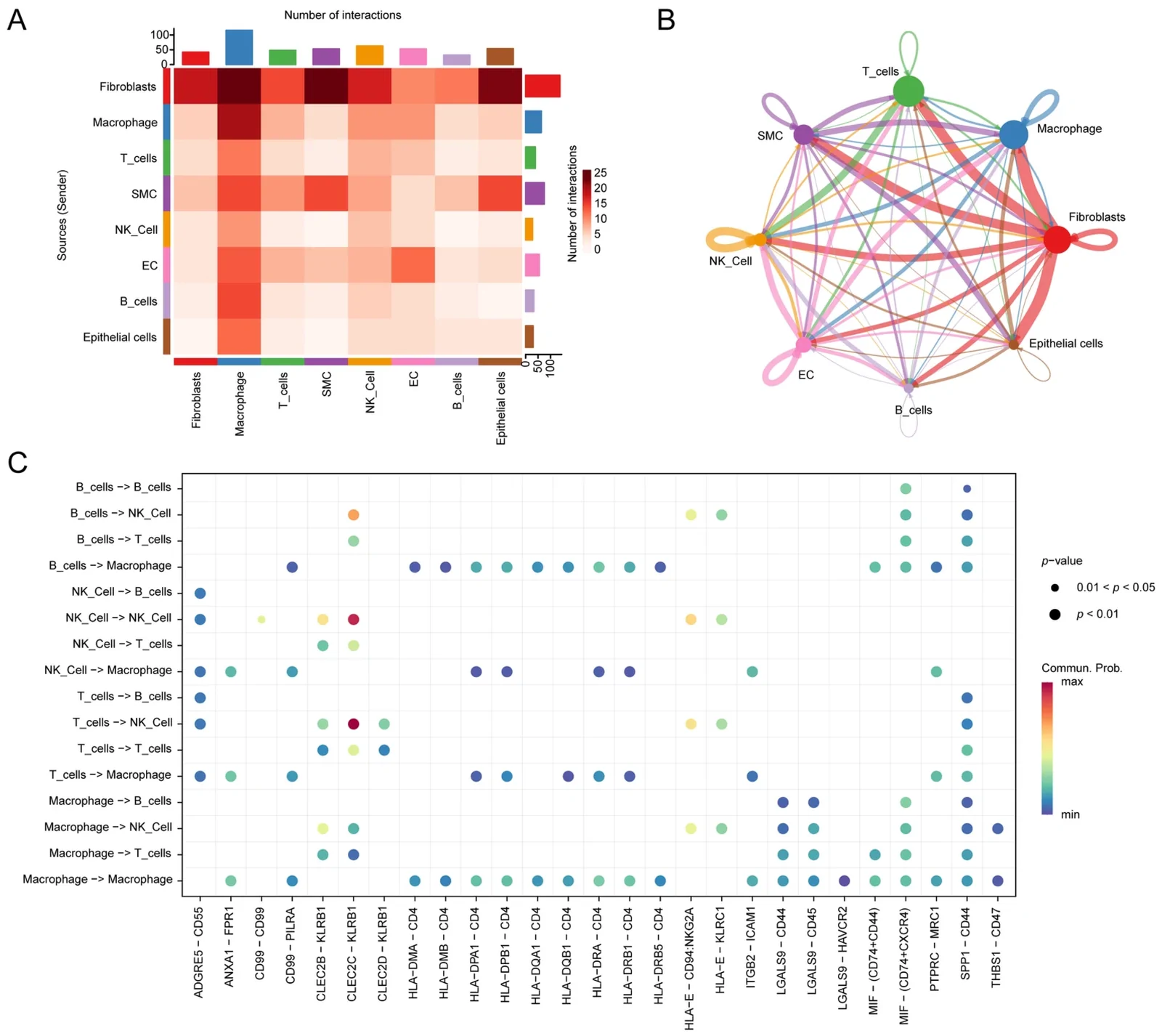
Cell–cell communication analysis of the pulmonary vascular microenvironment. (**A**). Heatmap illustrating the abundance of ligand–receptor interaction pairs across eight distinct cell populations, where deeper red hues indicate a higher frequency of interactions. (**B**). Network topology depicting the magnitude of intercellular signaling among the eight cell types; nodes represent specific cell lineages, directed arrows signify the signaling trajectory from signal-emitting to signal-receiving cells, and edge thickness correlates proportionally with interaction strength. (**C**). Bubble plot characterizing ligand–receptor mediators governing communication within four immune subsets. The vertical axis identifies the participating cell types, while the horizontal axis lists specific ligand–receptor dyads. Circle area reflects statistical significance, with larger markers denoting a *p*-value < 0.01, and the color gradient transitioning from blue to red indicates an increasing probability of cellular crosstalk.

CellChat analysis inferred intercellular communication among the eight annotated cell types, with fibroblasts and macrophages showing relatively high numbers of inferred interactions (Figure 7A,B). Among ligand–receptor interactions involving the four immune-cell populations, the CLEC2C-KLRB1 interaction from T cells to natural killer cells showed relatively high communication strength (Figure 7C).

CytoTRACE analysis showed higher scores in fibroblasts, corresponding to a lower inferred differentiation state and greater differentiation potential (Figure S11A,B). Pseudotime analysis identified State 1 as the inferred trajectory origin, and *DCN* and *DKK1* showed relatively higher expression at earlier/low-pseudotime regions (Figure S11D–G). These findings suggest that the model genes may reflect dynamic cellular-state transitions during pulmonary vascular remodeling. Because trajectory inference across heterogeneous cell types is exploratory, these results should be interpreted as hypothesis-generating and require experimental validation.

### 2.11. Hemodynamic Parameters and Candidate Gene Identification in PH Models

**Figure 8:**
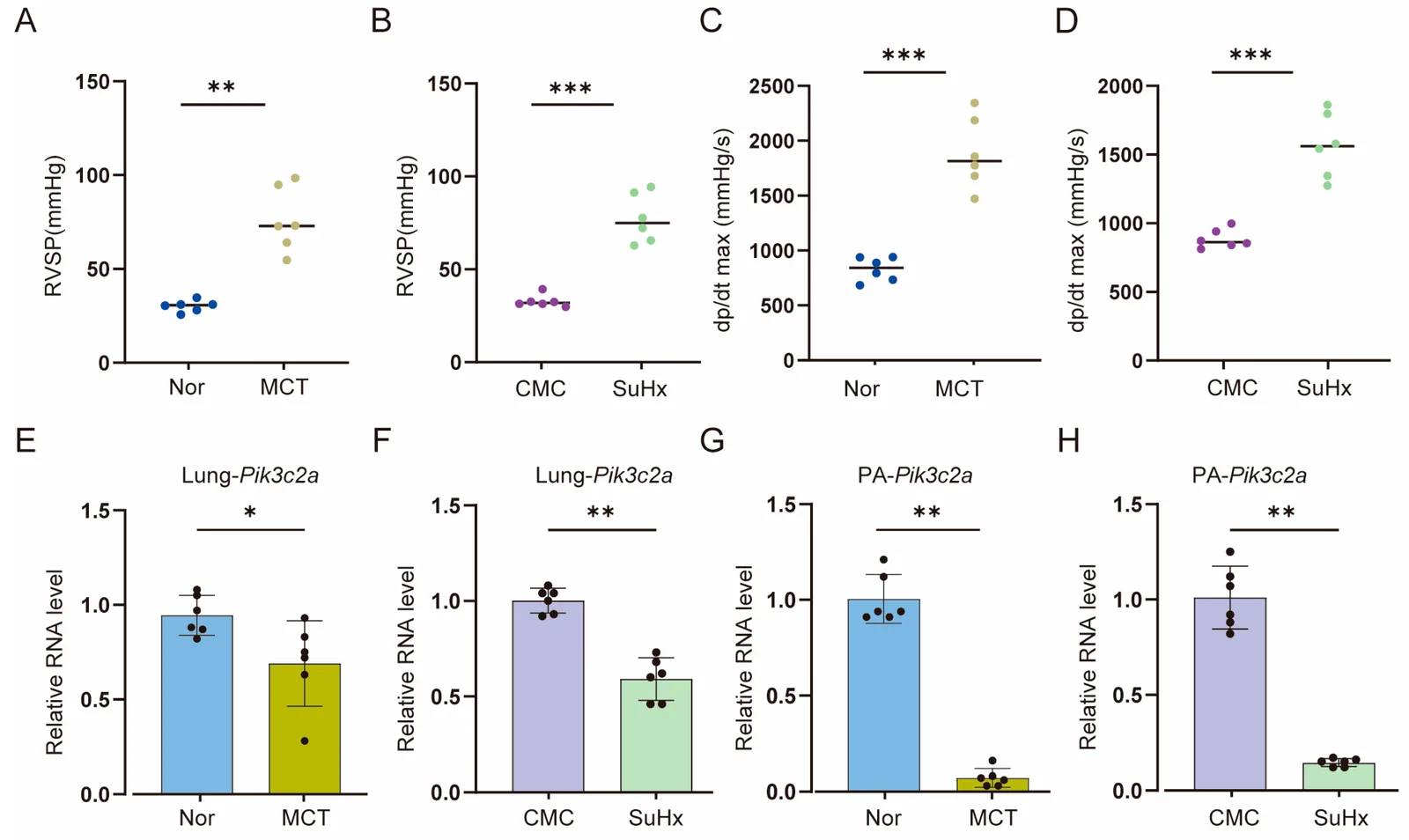
Hemodynamic assessment and *Pik3c2a* expression in two rat PH models. (**A**,**B**). RVSP in the MCT and SuHx rat PH models (Nor, *n* = 6; MCT, *n* = 6; CMC, *n* = 6; SuHx, *n* = 6). (**C**,**D**). dP/dt max in the MCT and SuHx rat PH models. (**E**,**F**). *Pik3c2a* expression in lung tissue assessed by qRT-PCR. (**G**,**H**). *Pik3c2a* expression in pulmonary arteries assessed by qRT-PCR. Data are presented as mean ± SEM, Individual data points are shown as black dots (*n* = 6 per group). Nor, normoxia control for the MCT model; MCT, monocrotaline-induced PH model; CMC, carboxymethyl cellulose vehicle control for the SuHx model; SuHx, Sugen5416 plus chronic hypoxia model; PA, pulmonary artery. Relative expression was normalized to β-actin. * *p* < 0.05, ** *p* < 0.01, *** *p* < 0.001.

RVSP was higher in the MCT and SuHx groups than in their corresponding control groups (Nor, 30.17 ± 3.03; MCT, 65.08 ± 21.34; CMC, 32.82 ± 3.32; SuHx, 77.29 ± 13.13). Similarly, dP/dt max was increased in the PH model groups (Nor, 830.47 ± 108.43; MCT, 1654.09 ± 511.38; CMC, 886.22 ± 69.13; SuHx, 1566.77 ± 234.96) (Figure 8A–D; Table S7). Collectively, these hemodynamic changes supported the successful establishment of the two rat PH models.

To further validate the expression profiles of candidate genes *DKK1*, *PIK3C2A*, *UNC5B* and *DCN*, their expression levels were detected by qRT-PCR in two rat models of PH. *Pik3c2a* expression was significantly reduced in both lung tissue and pulmonary arteries from the MCT and SuHx rat PH models (Figure 8E–H). *Dcn* showed no significant expression changes in lung tissue of the two rat PH models (Figure S12A,B). The downregulated expression of *Unc5b* in the lung tissue of rat PH models was inconsistent with the previous analysis results (Figure S12C,D). *Dkk1* was significantly downregulated in pulmonary arteries from the MCT and SuHx models (Figure S12E,F). Based on the combined transcriptomic, single-cell, and rat-model findings, *PIK3C2A* was prioritized as the candidate gene for subsequent investigation.

## 3. Discussion

Through integrative analysis of human lung transcriptomic data, this study identified 43 ARDEGs, which were enriched in functional terms and pathways related to vascular development, collagen-containing extracellular matrix, focal adhesion, integrin binding, and AGE-RAGE signaling. Application of WGCNA together with three machine-learning feature-selection strategies converged on a four-gene signature comprising *DKK1*, *PIK3C2A*, *UNC5B* and *DCN*. Cross-species validation provided stronger support for *DCN* and *UNC5B* in GSE1909, whereas single-cell localization and subsequent rat-model validation provided complementary evidence that ultimately prioritized *PIK3C2A* for further investigation.

The enrichment profile of ARDEGs is consistent with the known multicellular pathology of PH. Angiogenesis-related genes were not limited to endothelial growth pathways [14]; instead, they converged on extracellular matrix [15], adhesion [16], transcriptional regulation [17], hypoxia [18], inflammation [19], and metabolic vascular pathways [20]. From a pathophysiological perspective, these enrichment patterns are consistent with known features of pulmonary vascular remodeling in PH, involving endothelial injury [21], aberrant activation of smooth muscle cells and fibroblasts [22], increased matrix stiffness, inflammatory cell infiltration [23], and dysregulated hypoxic signaling pathways. Of note, the observed reduction in the angiogenesis-related score in PH may reflect several non-mutually exclusive biological or compositional factors. It could reflect a progressive loss of homeostatic vascular maintenance signals, a maladaptive shift that overrides endogenous reparative attempts, or alternatively, it may partly reflect altered cellular composition in bulk lung tissue. To differentiate between these possibilities, further validation is warranted using samples obtained at earlier disease stages or through cell-sorting approaches that enable enrichment of specific vascular wall cellular compartments.

*DCN* (decorin) encodes decorin, a small leucine-rich proteoglycan involved in extracellular matrix organization and growth-factor modulation [24]. Its fibroblast localization suggests that the bulk *DCN* signal may mark adventitial or stromal matrix remodeling rather than endothelial angiogenesis alone [25]. Reduced DCN protein expression in the right ventricle has been reported in several experimental PH models, including PAB, MCT, and SuHx [26]. *UNC5B* (Unc-5 Netrin Receptor B), a receptor of the Netrin family, plays a pivotal role in both neural and vascular development [27]. In vascular endothelial cells, *UNC5B* has been reported to restrain endothelial proliferation and migration and to contribute to junctional stability under laminar shear stress [28]. In GSE1909, *DCN* showed strong discriminative performance (AUC > 0.9), whereas *UNC5B* showed moderate-to-high discriminative performance (0.7 < AUC < 0.9); however, their expression patterns were not consistently reproduced in the subsequent rat PH models. The discordance between GSE1909 and the rat-model experiments may reflect differences in species and experimental context, including the hypoxia-based design and post-hypoxia comparison group used in GSE1909. These findings therefore highlight cross-species heterogeneity rather than providing uniform validation of *DCN* and *UNC5B* across PH models.

*DKK1* (Dickkopf-1) is a secreted glycoprotein and a well-established classic inhibitor of the Wnt/β-catenin signaling pathway [29]. The role of *DKK1* in PH has received increasing attention in recent years. In the present study, we found that *DKK1* expression was downregulated in lung tissues from PH patients and exhibited favorable discriminative performance (AUC = 0.944). Subsequent detection in pulmonary arteries from rat PH models revealed that *DKK1* expression was decreased in both the MCT and SuHx models, consistent with the dataset analysis. However, previous studies have reported that hypoxia can induce DKK1 expression and that increased DKK1 may promote endothelial proliferation and reduce apoptosis through the AKT–Sp1–SHMT2 pathway [30], thereby driving PH pathogenesis. Notably, other studies have reported significant upregulation of DKK1 in the serum and right ventricular myocardial tissues of MCTinduced PH rats [31]. Together with previous reports, our findings indicate that *DKK1* expression in PH may vary according to tissue compartment and disease context. This context-dependent pattern may explain the differences observed between whole-lung transcriptomic data and individual experimental PH models and warrants cell-type-specific investigation.

The protein encoded by the *PIK3C2A* gene is a Class II member of the phosphatidylinositol 3-kinase (PI3K) family, designated as PI3K-C2α, and exhibits broad expression across human tissues [32]. The enzymatic activity of *PIK3C2A* is essential for multiple cellular processes, including primary cilium assembly [33], clathrin-mediated endocytosis [34], and intracellular vesicular transport [35]. *PIK3C2A* has been established as a critical regulator of angiogenesis and endothelial barrier integrity [36]. *PIK3C2A* has also been implicated in VSMC phenotype switching and autophagy regulation [37]. Endothelial cells and vascular smooth muscle cells are major cellular contributors to pulmonary vascular remodeling in PH [38]. Our single-cell analysis (GSE210248) revealed that *PIK3C2A* is highly expressed in endothelial cells, with additional expression in fibroblasts and smooth muscle cells, suggesting cell-type-specific roles in vascular homeostasis. *PIK3C2A* was highly expressed in endothelial cells in GSE210248 and was downregulated in pulmonary arteries from the MCT and SuHx models. Together with previous evidence linking PI3K-C2α to endothelial barrier integrity and VE-cadherin trafficking [12,36], these findings support the possibility that altered *PIK3C2A* contributes to endothelial dysfunction during pulmonary vascular remodeling. However, its effects on barrier function, EndMT, intracellular signaling, and smooth-muscle-cell phenotype were not directly tested here and require functional validation.

Among the four model genes, *PIK3C2A* showed strong discriminative performance in GSE15197 (AUC = 0.906) and was preferentially expressed in endothelial cells in the GSE210248 single-cell dataset. Its performance was more limited in the murine GSE1909 dataset, and rat-model validation showed downregulation in the MCT and SuHx models. The more limited performance of PIK3C2A in GSE1909, together with its downregulation in the MCT and SuHx rat models, indicates context-dependent expression across species and experimental PH models. Such differences may reflect variation in species, tissue composition, disease context, or experimental design, but the underlying mechanisms were not examined in the present study. Functional studies will therefore be required to determine whether the manipulation of PIK3C2A has therapeutic relevance in PH. Therefore, the convergence of human transcriptomic screening, endothelial-cell localization, immune associations, and experimental support in two complementary PH models prioritized *PIK3C2A* as a candidate for further mechanistic investigation. Independent human cohorts and functional experiments will be required to establish its diagnostic and biological relevance.

Immune-infiltration and CellChat analyses further suggest an immune–stromal component of angiogenesis-related remodeling in PH. In HighScore samples, *PIK3C2A* correlated positively with activated mast cells (r = 0.917, *p* < 0.05) and negatively with M2 macrophages (r = −0.967, *p* < 0.05), while CellChat identified relatively frequent fibroblast-macrophage interactions and a prominent CLEC2C-KLRB1 interaction from T cells to natural killer cells. Because these analyses are inferential, the biological roles of these immune and cell–cell communication patterns require experimental validation.

A strength of this study is the integration of bulk transcriptomics, machine-learning prioritization, single-cell localization, and experimental validation across complementary PH models, allowing candidate genes to be evaluated from multiple biological perspectives. Several limitations should also be emphasized. First, the bioinformatic analyses were retrospective and relied on public datasets, and differences in tissue source, species, platform, PH subtype, and experimental context may contribute to heterogeneity across analyses. Second, the primary discovery dataset consisted of whole-lung tissue; therefore, the four-gene signature should be regarded as a tissue-discriminating signature rather than a validated blood-based or non-invasive diagnostic test. Third, the sample size is limited, and machine-learning models may overfit without independent human validation. Fourth, the scRNA-seq dataset included only three PAH and three control donors, which limits the robustness of between-group cellular comparisons. Fifth, the study is currently limited to candidate gene screening and expression validation; it has not yet explored the mechanistic basis by which the target genes exert their effects on the progression of PH.

Future studies should determine the functional role of *PIK3C2A* in pulmonary arterial endothelial cells using gain- and loss-of-function approaches, with particular attention to angiogenic capacity, endothelial barrier function, and EndMT. Its causal relevance to pulmonary vascular remodeling should then be evaluated through targeted manipulation in complementary PH models such as MCT and SuHx. Finally, independent human tissue and blood cohorts will be required to determine whether *PIK3C2A* has reproducible diagnostic or prognostic value in PH.

## 4. Materials and Methods

### 4.1. Data Acquisition and Study Design

Publicly available datasets from the Gene Expression Omnibus (GEO; https://www.ncbi.nlm.nih.gov/geo/ accessed on 20 January 2026) [39] were used. GSE15197 [40] and GSE1909 [41] were downloaded using the R package GEOquery (version 2.70.0) [42]. GSE15197 (GPL6480) comprised lung samples from 18 PAH subjects and 13 normal controls and served as the primary human bulk–transcriptomic discovery dataset. GSE1909 (GPL1674) comprised 20 hypoxia-induced murine PH lung tissue samples and 16 post-hypoxia comparison group samples, which were collected from mice after 21 days of chronic hypoxic exposure and subsequent return to normoxia; on days 22, 24, 28, and 35, mouse genes were converted to human orthologs using the homologene function for cross-species analyses. GSE210248 (GPL20301) comprised human pulmonary-artery scRNA-seq data from three PAH and three control samples [43,44]. Dataset information is summarized in Table 2. In this study, the human datasets comprised samples from patients with PAH, whereas the animal datasets represented experimental PH induced by hypoxia, MCT, or SuHx. For cross-cohort and cross-species analyses, the integrated disease phenotype was uniformly referred to as PH.

The study workflow consisted of bulk-transcriptome preprocessing, differential-expression analysis, intersection with curated angiogenesis-related genes, GO/KEGG enrichment, WGCNA, angiogenesis-score construction, GSVA, immune-infiltration analysis, machine-learning feature selection, cross-species validation, protein-interaction analysis, single-gene GSEA, and scRNA-seq-based cell annotation, CellChat and pseudotime analyses, followed by experimental validation of candidate-gene expression in two rat PH models.

### 4.2. Angiogenesis-Related Gene Collection and Bulk Data Preprocessing

ARGs were retrieved from GeneCards (https://www.genecards.org/, accessed on 25 January 2026) [45] using the keyword “Angiogenesis”; only protein-coding genes with a relevance score > 3 were retained, yielding 570 genes. An additional 510 ARGs were obtained from a previously published study [46]. After merging these two sets and removing duplicates, 874 unique ARGs were retained. Bulk microarray data from GSE15197 and GSE1909 were normalized and processed using the R package limma (version 3.62.1) [47]. The expression distributions before and after normalization were evaluated using boxplots (Figure S2). For GSE1909, data were downloaded using GEOquery, and probes were mapped to mouse gene symbols according to the platform annotation. The expression values of probes mapped to the same gene were averaged and normalized. Subsequently, mouse genes were converted to human orthologs using homologene, and duplicate human ortholog mappings were merged. After processing, 2137 available human ortholog genes were obtained and intersected with the 874 ARGs, yielding a candidate ARG set of 161 genes for cross-species comparison (Table S1). This intersection step was based solely on the mappability and detectability of genes in GSE1909 and did not use sample-group assignments or differential-expression results.

### 4.3. Differential Expression and Enrichment Analyses

Using GSE15197, we compared PH and control samples with the R package limma (version 3.62.1) to detect DEGs. DEGs were called if |log2FC| > 0.5 and nominal *p* < 0.05. For each gene, both the nominal *p* value and the Benjamini–Hochberg (BH)-adjusted *p* value were calculated. DEGs were called if |log2FC| > 0.5 and nominal *p* < 0.05. These criteria were used for exploratory candidate screening, whereas BH-adjusted *p* values were not used as inclusion criteria for DEG selection in this study. Both nominal and adjusted *p* values were retained and reported to distinguish exploratory findings from multiple-testing-controlled results. ARDEGs represented the overlap between exploratory DEGs and ARGs. Volcano plots and Venn diagrams were used to show differential expression and intersection results, respectively; ARDEG expression patterns were displayed using pheatmap (version 1.0.12).

ARDEGs were subjected to GO [48] and KEGG [49] enrichment analyses in clusterProfiler (version 4.10.0) [50]. BP, CC, MF, and KEGG pathway categories were examined. We regarded terms as significant when the Benjamini–Hochberg-adjusted *p* was <0.05 and the q value was <0.25. After ranking by adjusted *p* value, the five most significant terms per category were visualized with bubble plots and gene-network plots.

### 4.4. Weighted Gene Co-Expression Network Analysis

WGCNA [51,52] was performed using the 50% most variable genes from GSE15197. The soft-thresholding power was selected by evaluating the scale-free topology model fit and mean connectivity. A power of 16 was chosen because it yielded a scale-free topology fit index of 0.85. The minimum module size was set to 100 genes, and modules with highly similar expression profiles were merged using a module-merging cut height of 0.2. Module eigengenes (MEs) were calculated and correlated with the PH/control phenotype. After excluding the gray module, modules with an absolute module–phenotype correlation coefficient |r| > 0.3 and *p* < 0.05 were considered phenotype-associated modules. No unified multiple-testing or FDR correction was applied to the module–phenotype correlation analyses. These modules were ranked according to the absolute value of their module–phenotype correlation coefficients, and the top-ranked module, MEblue (|r| = 0.753, nominal *p* = 1.03 × 10^−6^), was selected as an exploratory candidate module for subsequent analyses. The genes in the selected module were then intersected with ARDEGs to obtain the candidate genes.

Gene significance (GS) and module membership (MM) were calculated and visualized to characterize the correlations of individual module genes with the PH/control phenotype and the corresponding module eigengene, respectively. However, no hard thresholds for GS or MM were applied for gene selection. The GS/MM analyses were treated as exploratory associations and were not subjected to unified multiple-testing or FDR correction. Thus, candidate-gene identification was based on the combined evidence of module-level phenotype association and the intersection between genes in the selected module and ARDEGs, rather than on GS/MM threshold-based filtering.

### 4.5. Angiogenesis Score Construction and Pathway Activity Analysis

An angiogenesis score (A.score) was calculated for each GSE15197 sample using ssGSEA implemented in the R package GSVA (version 1.50.0), based on the module genes [53,54]. The score was compared between PH and control samples, and its discriminative performance was evaluated by ROC analysis using the R package pROC (version 1.18.5). PH samples were further divided into HighScore and LowScore groups according to the median A.score. Module gene expression was compared between the two groups, and ROC curves were generated to evaluate the ability of individual module genes to distinguish HighScore from LowScore PH samples.

GSVA was performed using the R package GSVA (version 1.50.0) with the c2.cp.v2023.2.Hs.symbols gene-set collection from MSigDB [55] to identify pathway-level differences between HighScore and LowScore PH samples. Pathways with *p* < 0.05 were ranked by log fold change, and the top 10 positively and top 10 negatively enriched pathways were selected for visualization.

### 4.6. Immune Infiltration Analysis

The CIBERSORT algorithm was employed to quantify the relative abundance of 22 distinct immune cell subsets within the GSE15197 dataset [56]. Immune-cell composition was compared between HighScore and LowScore PH samples using the Wilcoxon rank-sum test. For each CIBERSORT-based group comparison, nominal *p* values were adjusted for multiple comparisons using the Holm method, and Holm-adjusted *p* values were used to assess the statistical significance. Spearman correlations among immune cells and between immune cells and module genes were calculated, and module gene–immune-cell associations with nominal *p* < 0.05 were retained for visualization. As a complementary analysis, ssGSEA was used to estimate the relative abundance of 28 curated immune-cell signatures [57] in GSE15197. Immune-cell abundance was compared between PH and control samples using the Wilcoxon rank-sum test. The resulting nominal *p* values were adjusted using the Holm method across the 28 immune-cell signatures, and Holm-adjusted *p* values were used for inference. Spearman correlations among immune-cell signatures and between immune-cell signatures and model genes were visualized using ggplot2 (version 3.4.4) and pheatmap (version 1.0.12).

### 4.7. Machine Learning Feature Selection and Validation

Module genes were evaluated using three complementary approaches: logistic regression, RF, and LASSO regression. For univariate logistic regression, each gene was analyzed separately, with PH status as the dependent variable and the expression level of the corresponding gene as the independent variable. Odds ratios (ORs), 95% confidence intervals (CIs), and *p* values were estimated using binomial logistic regression. Genes with *p* < 0.05 were considered to be significantly associated with PH status and were retained as statistically associated candidates. LASSO regression was performed using the R package glmnet [58] with set.seed(2024) and family = “binomial”. Ten-fold cross-validation was used to determine the penalty parameter, and the value of λ yielding the minimum cross-validated error (lambda.min) was selected as the optimal penalty parameter. The genes with non-zero coefficients at lambda.min were retained as the LASSO-selected genes. RF analysis was performed using the randomForest package [59] with set.seed (2024) and ntree = 1000. The optimal number of variables was determined according to the lowest average error rate obtained from five repetitions of 10-fold cross-validation. In this analysis, the lowest average error rate corresponded to all 16 input variables. Therefore, RF did not further reduce the number of genes; instead, all 16 genes were ranked according to their MeanDecreaseGini importance values. Thus, dimensionality reduction was primarily achieved by LASSO regression. The final four-gene set consisted of the genes selected by LASSO at lambda.min, all of which also met the significance criterion in univariate logistic regression and were included in the RF importance ranking. These genes were defined as the candidate model genes.

The expression levels and ROC performance of model genes were evaluated in GSE15197. Cross-species validation was performed in GSE1909 after mouse-to-human ortholog mapping.

### 4.8. Protein Interaction and Single-Gene GSEA Analyses

GeneMANIA (https://genemania.org/, accessed on 18 February 2026) [60] was used to construct a functional-association network linking the model genes with predicted functionally related partners. For single-gene GSEA, all genes in GSE15197 were ranked according to their correlation with each model gene, followed by enrichment analysis using clusterProfiler (version 4.10.0) and the c2.all.v2024.1.Hs.symbols gene set collection from MSigDB. Gene sets containing 10–500 genes were evaluated, and terms with adjusted *p* < 0.05 and q value < 0.05 after Benjamini–Hochberg correction were considered significant.

### 4.9. Assessment of Discriminative Capacity for Model Genes

To validate the model genes, their expression levels were compared between PH and the corresponding comparison groups in GSE15197 and GSE1909. Receiver operating characteristic (ROC) curves were generated using the R package pROC (version 1.18.5), and the area under the curve (AUC) was calculated to evaluate the ability of each model gene to discriminate PH from the corresponding comparison group. GSE1909 was used for cross-species validation after mouse-to-human ortholog mapping. In this study, the 95% CI of the AUC was calculated using the ci.auc function in the pROC package. Since no cut-off was prespecified in this study, the sensitivity and specificity corresponding to a cut-off were not estimated.

### 4.10. Single-Cell RNA-Seq Analysis

The GSE210248 scRNA-seq dataset was processed using Seurat v5.0. A Seurat object was created by retaining genes detected in at least three cells and cells with at least 200 detected genes. Cells with nCount_RNA > 10,000, nFeature_RNA > 3000, log10GenesPerUMI < 0.8, or percent.mt > 0.05 were excluded during quality control. After filtering, 21,730 cells were retained for downstream analysis.

Six samples were separately created as Seurat objects. After labeling the samples, they were merged into a single object with integrated expression data, followed by quality control and standard preprocessing. Gene expression data were normalized using the NormalizeData function with the LogNormalize method. Highly variable genes were identified using FindVariableFeatures with the variance-stabilizing transformation (VST) method, and the top 2000 highly variable genes were selected. The normalized data were scaled using ScaleData, followed by principal component analysis (PCA). Batch correction was performed using Harmony with the sample as the group.by.vars, and it was specified that FindNeighbors, FindClusters, and RunUMAP were all based on the first 30 Harmony dimensions, with a clustering resolution of 0.8. The first 30 Harmony dimensions were used to construct a shared nearest-neighbor graph with FindNeighbors and to perform UMAP dimensionality reduction using RunUMAP. Graph-based clustering was performed using FindClusters with a resolution parameter of 0.8. Cell types were assigned using SingleR and further validated based on the expression of canonical marker genes, including PECAM1, CLDN5, and VWF for endothelial cells; CD2, CD3D, and IL7R for T cells; LYZ, C1QA, and C1QB for macrophages; GNLY, PRF1, and KLRD1 for natural killer cells; MYH11, RGS5, and PLN for smooth muscle cells; CD79A, CD79B, and MS4A1 for B cells; SFTPB and SFTPC for epithelial cells; and FBLN1 and DCN for fibroblasts.

Feature plots and dot plots were generated to map *DCN*, *DKK1*, *PIK3C2A*, and *UNC5B* across major cell types. Cell–cell communication was inferred using CellChat (version 2.1.1) together with the CellChatDB.human ligand–receptor database [61]. Significant ligand–receptor interactions were inferred from interaction probabilities and permutation-based testing, and communication networks were summarized according to interaction number and strength. CytoTRACE [62] was used to estimate cellular differentiation potential, and pseudotime trajectories were reconstructed using monocle (version 2.30.1) [63,64,65] to characterize cellular-state transitions and model-gene expression dynamics. These single-cell analyses were interpreted as being hypothesis-generating because of the limited number of biological samples.

### 4.11. Establishment of Two Rat PH Models and Sample Collection

This study established two well-characterized rat models of PH, with six rats assigned to each experimental group (Nor group, *n* = 6; MCT group, *n* = 6; CMC group, *n* = 6; SuHx group, *n* = 6). Male Sprague–Dawley rats, aged 6–8 weeks and with an initial body weight range of 180–220 g, were housed under standard specific-pathogen-free (SPF) conditions with a 12 h light/dark cycle and ad libitum access to food and water. All rats were randomly allocated to four experimental groups using a computer-generated random number table, and all investigators performing hemodynamic measurements, tissue dissection, and subsequent qRT-PCR analyses were blinded to the group assignment. The Nor group served as the normoxic control for the MCT group, received an equal volume of phosphate-buffered saline (PBS) via subcutaneous injection, and was housed continuously under normoxic conditions (20% O_2_) for the full 5-week experimental duration. The MCT group received a single subcutaneous injection of monocrotaline (MCT) at a dose of 60 mg/kg, and was subsequently maintained under normoxic conditions (20% O_2_) for 3 weeks. For the SuHx group, rats first received a single subcutaneous injection of SU5416 at 10 mg/kg, followed by 21 consecutive days of chronic hypoxia exposure in a sealed, continuously flushed hypoxic chamber maintained at a stable oxygen concentration of 10% O_2_, with CO_2_ concentration controlled below 0.5% and humidity maintained at 50–60% to prevent CO_2_ accumulation and excessive dryness. After the 21-day hypoxic exposure, the SuHx rats were returned to normal room air (20% O_2_) for a 14-day reoxygenation period. As the strict vehicle control for the SuHx group, the CMC vehicle group received a single subcutaneous injection of an equal volume of carboxymethyl cellulose (CMC), the solvent vehicle used to dissolve SU5416, followed by the identical 21-day chronic hypoxia exposure (10% O_2_) and subsequent 14-day normoxic housing period. After the full model establishment schedule, right ventricular systolic pressure (RVSP) and the maximum rate of right ventricular pressure rise (dp/dt max) were invasively measured in all experimental rats via a right ventricular catheter. After hemodynamic assessment, the rats were euthanized and perfused with ice-cold PBS via the right ventricle. The main pulmonary artery and left lung tissues were carefully dissected, immediately snap-frozen in liquid nitrogen, and stored at −80 °C.

### 4.12. Quantitative Real-Time Polymerase Chain Reaction

The total RNA was extracted from lung tissues and pulmonary arteries using TRIzol reagent (Invitrogen, Carlsbad, CA, USA) according to the manufacturer’s instructions. RNA purity and concentration were assessed with a NanoDrop 2000 spectrophotometer (Thermo Fisher Scientific, Waltham, MA, USA), and RNA integrity was verified by 1% agarose gel electrophoresis. An equal amount of total RNA (1 μg) was reverse-transcribed into complementary DNA (cDNA) using PrimeScript RT Master Mix (Takara, Dalian, Liaoning, China, RR036A) within a 20 μL reaction system. The reverse-transcription reaction proceeded at 37 °C for 15 min and was halted by heat inactivation at 85 °C for 5 s. The resulting cDNA products were diluted to a final concentration of 100 ng/μL and stored at −20 °C until quantitative PCR assessment. Gene expression profiling for candidate targets in PH was conducted via qRT-PCR on a CFX Opus 96 instrument (Bio-Rad Laboratories, Hercules, CA, USA), incorporating TB Green^®^ Premix Ex Taq^TM^ II (Takara, Dalian, Liaoning, China, RR820A) alongside gene-specific primers detailed in Table S6. The cycling conditions were as follows: initial denaturation at 95 °C for 30 s, followed by 40 cycles of 95 °C for 5 s and 60 °C for 30 s, and a final melting curve analysis from 60 °C to 95 °C. For data normalization, β-actin served as the endogenous control, given its established stability across numerous PH investigations [66]. The relative abundance of target transcripts was quantified applying the 2^−ΔΔCt^ computational approach. Six biological samples were included in each experimental group, and each biological sample (lung tissue or pulmonary artery) was loaded in three replicate wells on the qPCR plate.

### 4.13. Statistical Analysis

Statistical analyses and bioinformatic workflows were performed using R (version 4.3.3), whereas qRT-PCR data and other experimental results were analyzed using GraphPad Prism 8.0. Data normality was assessed using the Shapiro–Wilk test and visual inspection of Q–Q plots. Given the relatively small sample size in some experiments (*n* = 6 per group), normality assessments were interpreted cautiously. For comparisons between two independent groups, normally distributed data were analyzed using a two-tailed unpaired Student’s *t*-test, whereas the Mann–Whitney U test was used for data that did not follow a normal distribution. Differences among three or more groups were evaluated using the Kruskal–Wallis test. Associations between molecular variables were examined using Spearman’s rank correlation analysis. qRT-PCR data are presented as the mean ± standard error of the mean (SEM). Unless otherwise specified, all tests were two-sided, and *p* < 0.05 was considered statistically significant.

## 5. Conclusions

This study identified an angiogenesis-related four-gene signature comprising *DCN*, *DKK1*, *PIK3C2A*, and *UNC5B* and characterized its pathway, immune, and cellular context in PH. Among the four core genes, *PIK3C2A* emerged as the most promising candidate, exhibiting consistently downregulated expression in both lung tissues and pulmonary arteries across two independent PH models (MCT and SuHx). These findings support further mechanistic and independent clinical evaluation of *PIK3C2A* as a candidate molecule associated with pulmonary vascular remodeling in PH.

## Figures and Tables

**Table 1:** Results of GO and KEGG enrichment analysis for ARDEGs.

Ontology	ID	Description	Gene Ratio	Bg Ratio	*p* Value	*p*.adjust	q Value
BP	GO:0045765	regulation of angiogenesis	14/43	360/18,986	2.88 × 10^−14^	4.66 × 10^−11^	2.40 × 10^−11^
BP	GO:1901342	regulation of vasculature development	14/43	369/18,986	4.04 × 10^−14^	4.66 × 10^−11^	2.40 × 10^−11^
BP	GO:0045766	positive regulation of angiogenesis	11/43	189/18,986	3.09 × 10^−13^	2.12 × 10^−10^	1.10 × 10^−10^
BP	GO:1904018	positive regulation of vasculature development	11/43	192/18,986	3.68 × 10^−13^	2.12 × 10^−10^	1.10 × 10^−10^
BP	GO:0043434	response to peptide hormone	12/43	446/18,986	1.93 × 10^−10^	8.93 × 10^−8^	4.60 × 10^−8^
CC	GO:0062023	collagen-containing extracellular matrix	8/43	430/19,960	3.25 × 10^−6^	4.95 × 10^−4^	3.46 × 10^−4^
CC	GO:0005925	focal adhesion	7/43	424/19,960	3.09 × 10^−5^	1.80 × 10^−3^	1.259 × 10^−3^
CC	GO:0030055	cell–substrate junction	7/43	434/19,960	3.59 × 10^−5^	1.80 × 10^−3^	1.259 × 10^−3^
CC	GO:0034774	secretory granule lumen	6/43	324/19,960	6.42 × 10^−5^	1.80 × 10^−3^	1.259 × 10^−3^
CC	GO:0060205	cytoplasmic vesicle lumen	6/43	329/19,960	6.99 × 10^−5^	1.80 × 10^−3^	1.259 × 10^−3^
MF	GO:0001221	transcription coregulator binding	6/43	117/18,737	2.63 × 10^−7^	5.39 × 10^−5^	3.29 × 10^−5^
MF	GO:0005178	integrin binding	6/43	161/18,737	1.72 × 10^−6^	1.76 × 10^−4^	1.08 × 10^−4^
MF	GO:0061629	RNA polymerase II-specific DNA-binding transcription factor binding	7/43	349/18,737	1.33 × 10^−5^	9.06 × 10^−4^	5.53 × 10^−4^
MF	GO:0001228	DNA-binding transcription activator activity, RNA polymerase II-specific	7/43	480/18,737	1.00 × 10^−4^	3.805 × 10^−3^	2.325 × 10^−3^
MF	GO:0001216	DNA-binding transcription activator activity	7/43	487/18,737	1.10 × 10^−4^	3.805 × 10^−3^	2.325 × 10^−3^
KEGG	hsa05205	Proteoglycans in cancer	11/38	204/9356	2.91 × 10^−10^	5.56 × 10^−8^	2.17 × 10^−8^
KEGG	hsa04933	AGE-RAGE signaling pathway in diabetic complications	8/38	101/9356	5.22 × 10^−9^	4.98 × 10^−7^	1.95 × 10^−7^
KEGG	hsa05417	Lipid and atherosclerosis	10/38	216/9356	9.39 × 10^−9^	5.98 × 10^−7^	2.34 × 10^−7^
KEGG	hsa05206	MicroRNAs in cancer	11/38	323/9356	3.66 × 10^−8^	1.75 × 10^−6^	6.84 × 10^−7^
KEGG	hsa04066	HIF-1 signaling pathway	7/38	110/9356	2.39 × 10^−7^	9.15 × 10^−6^	3.58 × 10^−6^

ARDEGs, angiogenesis-related differentially expressed genes; BP, biological process; CC, cellular component; MF, molecular function; KEGG, Kyoto Encyclopedia of Genes and Genomes.

**Table 2:** GEO microarray chip information.

	GSE15197	GSE1909	GSE210248
Platform	GPL6480	GPL1674	GPL20301
Species	Homo sapiens	Mus musculus	Homo sapiens
Tissue	lung	lungs	pulmonary artery
Samples in PH group	18	20	3
Samples in control group	13	16	3
Reference	PMID: 20081107	PMID: 15784693	PMID: 36099047

GEO, Gene Expression Omnibus; PH, pulmonary hypertension.

## Data Availability

The publicly available transcriptomic datasets analyzed in this study were obtained from the Gene Expression Omnibus (GEO) database (https://www.ncbi.nlm.nih.gov/geo/, accessed on 20 January 2026) under accession numbers GSE15197, GSE1909, and GSE210248. All data generated or analyzed during this study are included in this article and its Supplementary Information.

## Supplementary Materials

The following supporting information can be downloaded at https://www.mdpi.com/article/10.3390/ijms27219421/s1.
