# Supplementary material for: Machine Learning-Based Screening and Experimental Validation of PIK3C2A as a Candidate Angiogenesis-Related Molecule in Pulmonary Hypertension
Source: Int J Mol Sci. 2026 Oct 22;27(21):9421. doi: 10.3390/ijms27219421 (PMC13762966; doi:10.3390/ijms27219421)
Supplement: Supplementary file 1 [file ijms-27-09421-s001.zip › Supplementary Figures and Tables.pdf]

Supplementary Materials:

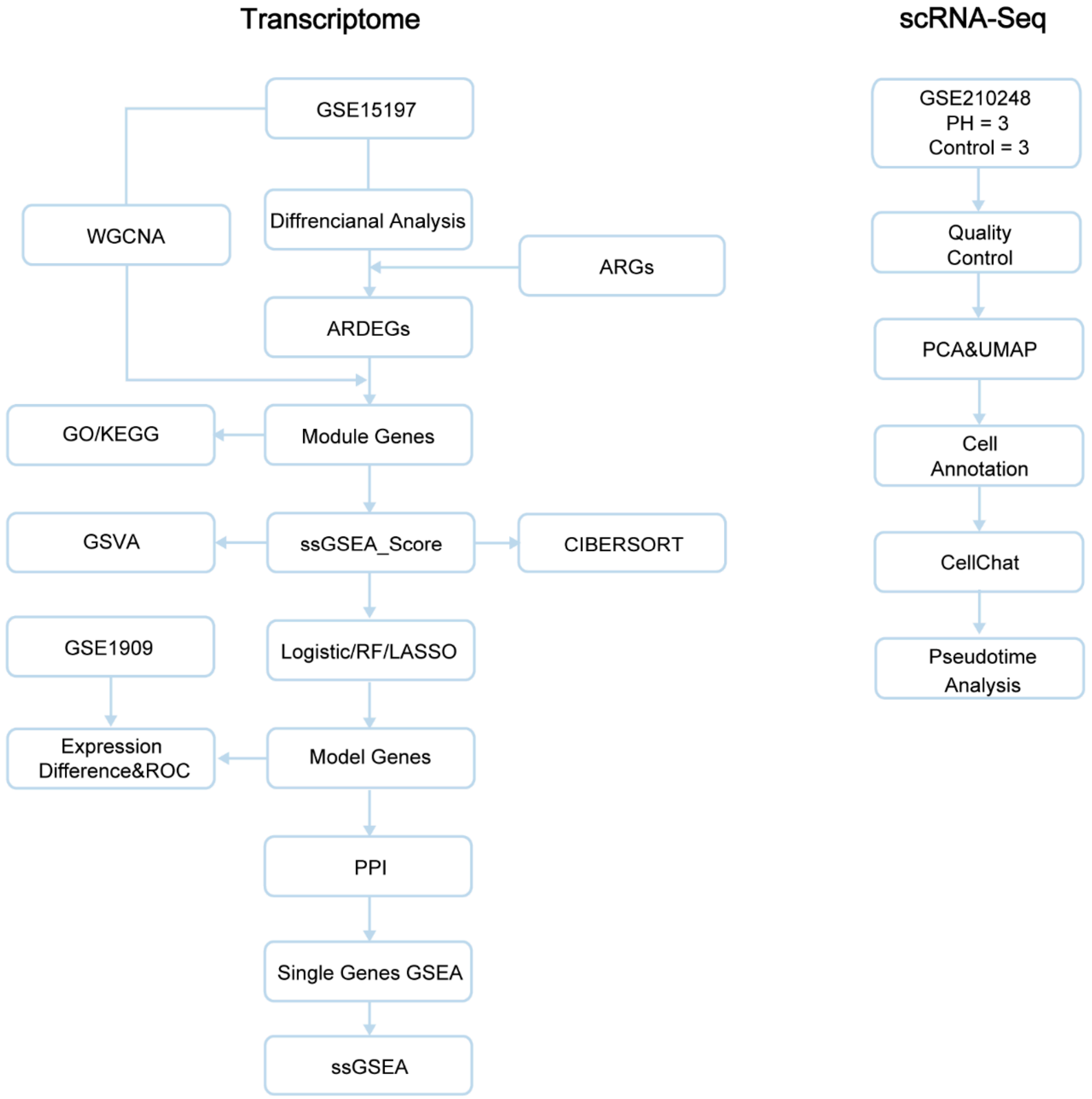

Figure S1. Flow Chart for the Comprehensive Analysis

ARGs, Angiogenesis Related Genes; ARDEGs, Angiogenesis Related Differentially Expressed Genes; WGCNA, Weighted Gene Co-expression Network Analysis; GSVA, Gene Set Variation Analysis; GO, Gene Ontology; KEGG, Kyoto Encyclopedia of Genes and Genomes; PPI, Protein-protein Interaction; RF, Random Forest; LASSO, Least Absolute Shrinkage and Selection Operator; ROC, Receiver Operating Characteristic; ssGSEA, Single-Sample Gene-Set Enrichment Analysis; PCA, Principal Component Analysis; UMAP, Uniform Manifold Approximation and Projection; PH, Pulmonary Hypertension.

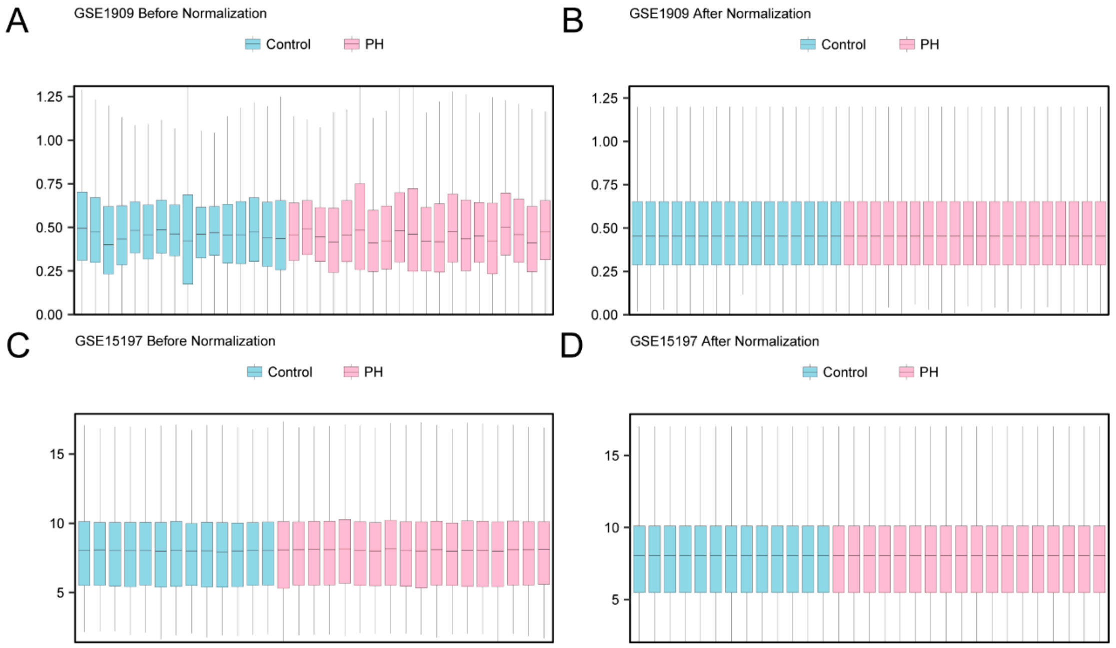

Figure S2. Normalization of TCC

A-B. Boxplots of the distribution of the pulmonary hypertension dataset GSE1909 before (A) and after (B) normalization. C-D. Boxplots of distribution of PH dataset GSE15197 before (C) and after (D) standardization. Pink is the PH group of the pulmonary hypertension dataset, and light blue is the Control group of the PH dataset.

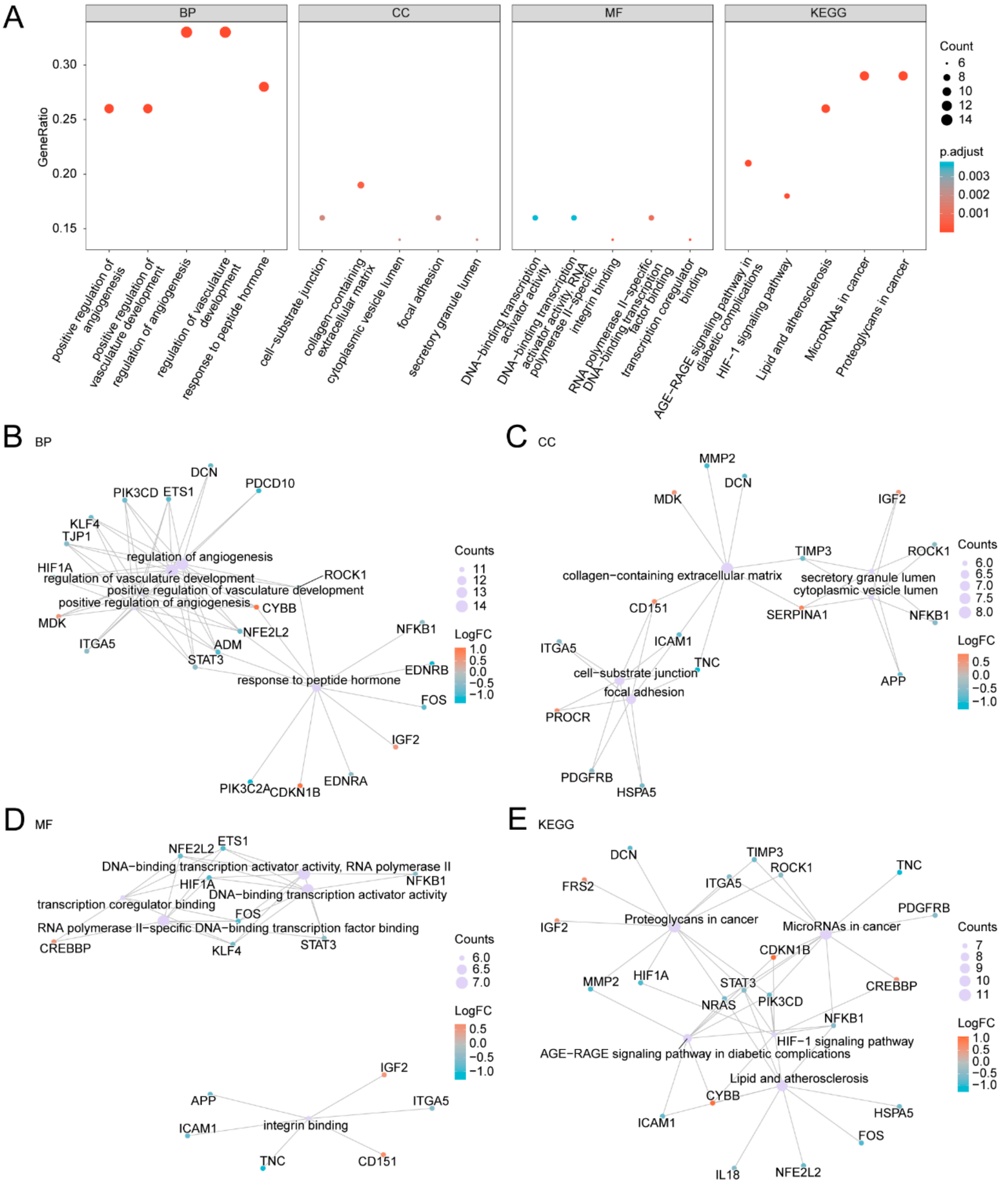

Figure S3. GO and KEGG Enrichment Analysis for ARDEGs

A. Bubble diagram illustrating the results of gene ontology (GO) and pathway (KEGG) enrichment analysis of ARDEGs. The x-axis represents the GO categories, which include biological process (BP), cellular component (CC), and molecular function (MF), as well as the biological pathways identified in the KEGG analysis. B-E. Gene ontology (GO) and pathway (KEGG) enrichment analysis results of angiogenesis-related differentially expressed genes (ARDEGs) network diagram showing: BP (B), CC (C), MF (D) and KEGG (E). Purple nodes correspond to individual entries,

with connecting lines illustrating the associations between these entries and their associated molecules. In the molecular nodes, a red coloration indicates higher LogFC values, signifying upregulation, while a blue coloration reflects lower LogFC values, indicating downregulation. In the bubble plot, the size of each bubble represents the number of genes involved, and its color indicates the magnitude of the adjusted p-value, where redder shades indicate smaller adjusted p-values and bluer shades indicate larger ones. The screening criteria for GO and KEGG enrichment analysis were p adjust < 0.05 and FDR value (q value) < 0.25, and the p value Correction method was Benjamini-Hochberg Correction (BH).

Table S4. GSVA results for high-score versus low-score PH samples

	logFC	AveExpr	t	P.Value	adj.P.Val	B
REACTOME_SARS_COV_2_TARGETS_PDZ_PROTEINS_IN_CELL_CELL_JUNCTION	1.116767	0.059004	7.096538	5.51E-08	0.000179	8.20252
KEGG_MEDICUS_PATHOGEN_HTLV_1_P12_TO_ANTIGEN_PROCESSING_AND_PRESENTATION_BY_MHC_CLASS_I_MOLECULES	0.909824	0.005298	3.733401	0.000758	0.027632	-0.52554
KEGG_MEDICUS_VARIANT_MUTATION_CAUSED_ABERRANT_SNCA_TO_PERK_ATF4_SIGNALING_PATHWAY	0.88845	0.006219	6.902593	9.44E-08	0.000179	7.715533
REACTOME_SYNTHESIS_OF_DIPHTHAMIDE_EEF2	0.826243	0.013902	5.310261	8.70E-06	0.003665	3.578004
KEGG_MEDICUS_VARIANT_MUTATION_CAUSED_ABERRANT_SOD1_TO_PERK_ATF4_SIGNALING_PATHWAY	0.788737	-0.01372	5.321712	8.41E-06	0.003665	3.608293
REACTOME_MASTL_FACILITATES_MITOTIC_PROGRESSION	0.777014	-0.06422	4.975797	2.27E-05	0.004823	2.69327
REACTOME_CYTOSOLIC_TRNA_AMINOACYLATION	0.767762	-0.05207	5.098032	1.60E-05	0.004543	3.016521
WP_DISORDERS_IN_KETONE_BODY_SYNTHESIS	0.764051	-0.06243	5.391313	6.89E-06	0.003665	3.792338
WP_KETONE_BODIES_SYNTHESIS_AND_DEGRADATION	0.764051	-0.06243	5.391313	6.89E-06	0.003665	3.792338
REACTOME_PROPIONYL_COA_CATABOLISM	0.753084	-0.08838	3.813396	0.000608	0.026521	-0.32515
KEGG_MEDICUS_REFERENCE_TRANSPORT_OF_CALCIUM	-0.64983	0.025465	-3.78764	0.000653	0.02692	-0.38982
REACTOME_LECTIN_PATHWAY_OF_COMPLEMENT_ACTIVATION	-0.6724	-0.00845	-4.37885	0.000125	0.01448	1.124535

KEGG_MEDICUS_REFERENCE_LECTIN_PATHWAY_OF_COAGULATION _CASCADE_PROTHROMBIN_TO_THROMBIN	-0.68243	0.001566	-4.62568	6.20E-05	0.010687	1.770275
BIOCARTA_PPARG_PATHWAY	-0.68645	-0.03112	-4.27805	0.000166	0.015025	0.862634
REACTOME_THE_FATTY_ACID_CYCLING_MODEL	-0.70944	0.073494	-3.89946	0.00048	0.024105	-0.10803
BIOCARTA_DICER_PATHWAY	-0.75287	0.06345	-2.80922	0.008502	0.092076	-2.70513
REACTOME_POST_TRANSCRIPTIONAL_SILENCING_BY_SMALL_RNAS	-0.79663	0.111189	-3.43072	0.001717	0.041219	-1.26954
KEGG_MEDICUS_REFERENCE_MANNOSE_TYPE_O_GLYCAN_BIOSYNTHESIS _POMT_TO_POMK	-0.80075	0.029588	-3.62073	0.00103	0.032284	-0.80526
REACTOME_FICOLINS_BIND_TO_REPETITIVE_CARBOHYDRATE _STRUCTURES_ON_THE_TARGET_CELL_SURFACE	-0.83243	-0.00334	-5.18931	1.23E-05	0.004245	3.25802
BIOCARTA_PELP1_PATHWAY	-0.85656	-0.03841	-5.25369	1.02E-05	0.003881	3.428356

GSVA，Gene Set Variation Analysis

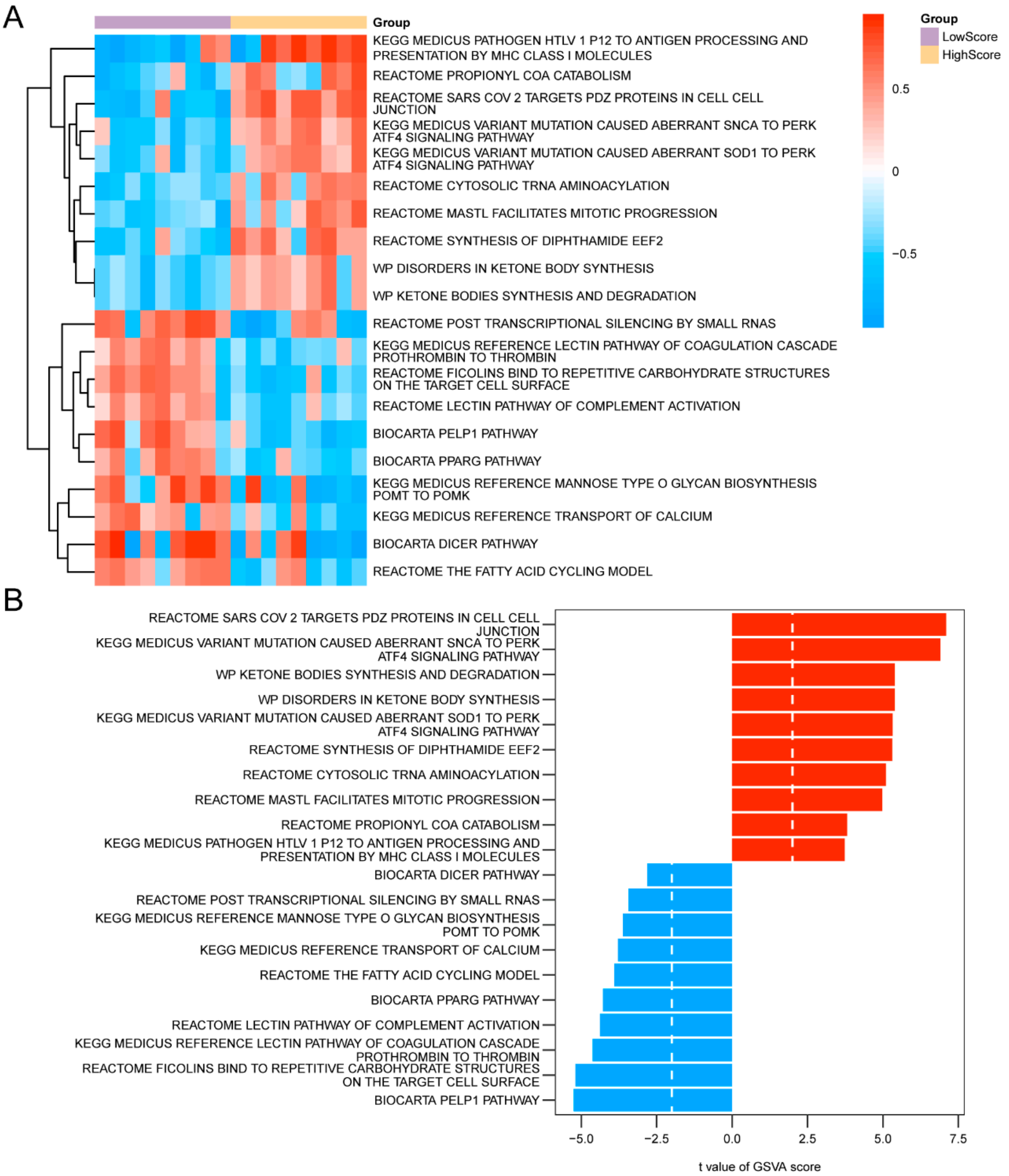

Figure S4. GSVA pathway analysis between high-score and low-score PH samples

A-B. Heat map (A) and bidirectional bar graph (B) of gene set variation analysis (GSVA) results between HighScore and LowScore of dataset GSE15197. Yellow for HighScore, purple for LowScore. GSVA was performed, and enrichment results were filtered using a significance threshold of $p < 0.05$. Differentially enriched pathways were ranked according to their logFC. The top 10 positively and top 10 negatively enriched pathways were selected for further visualization. In the accompanying heat map, red indicates high enrichment levels while blue represents low

enrichment levels.

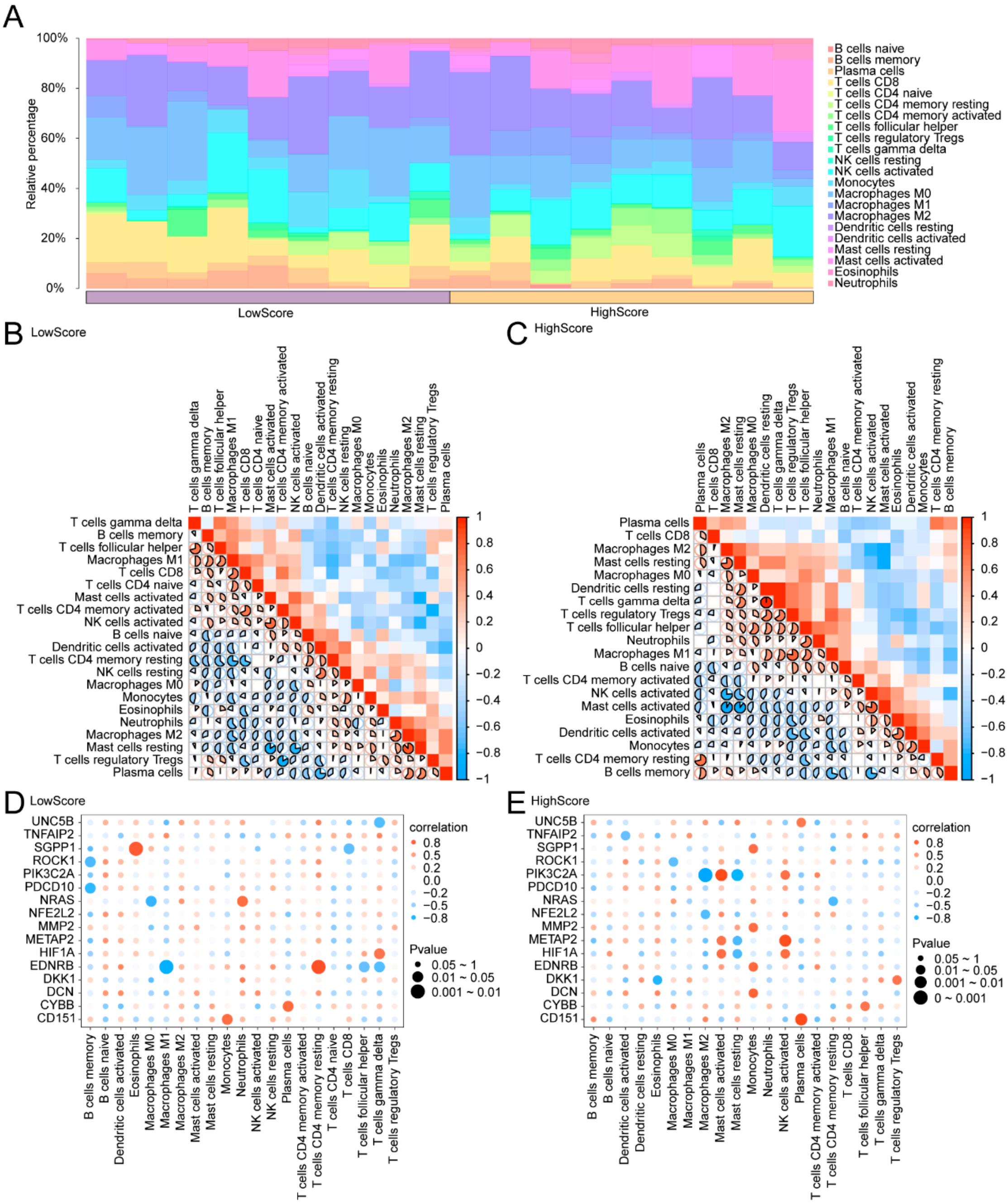

Figure S5. Risk Group Immune Infiltration Analysis by CIBERSORT Algorithm

A. Bar graph illustrating the relative proportions of infiltrating immune cells in PH patient samples, comparing the LowScore and HighScore groups. B-C show correlation analyses of immune cell infiltration abundance for the LowScore (B) and HighScore (C) subgroups of PH samples. D-E present bubble plots that depict the relationships between immune cell infiltration abundance and module genes in the LowScore (D) and HighScore (E) groups. Bubble sizes correspond to distinct ranges of nominal P values: 0.05-1, 0.01-0.05, 0.001-0.01, and 0-0.00

1, with larger bubbles indicating stronger correlations. Correlation coefficients (r values) are c ategorized as follows: absolute values below 0.3 indicate weak or no correlation; 0.3–0.5 indi cate weak correlation; 0.5–0.8 indicate moderate correlation; and above 0.8 indicate strong co rrelation. The HighScore group is represented in yellow, while the LowScore group is shown in purple. In the bubble plots, yellow indicates positive correlations, green signifies negative correlations, and color intensity reflects the strength of the correlation.

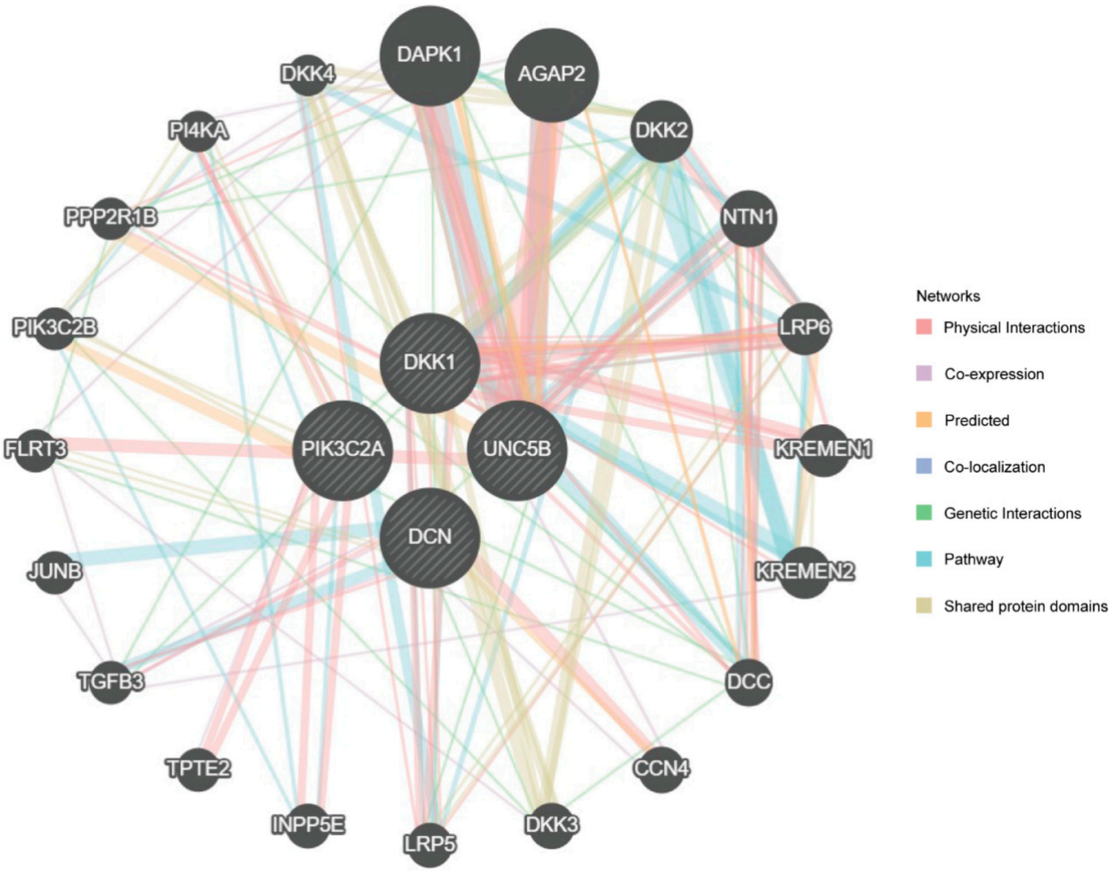

Figure S6. PPI Network Analysis

The GeneMANIA website was utilized to predict the interaction network of model genes and their functionally related counterparts. In the resulting diagram, circles represent the model genes along with their functionally similar genes, while the colors of the connecting lines indicate different types of functional associations. PPI, Protein-protein Interaction Network.

Table S5. Single-gene GSEA results for model genes

Gene	ID	setSize	enrichmentScore	NES	pvalue	p.adjust	qvalue
DKK1	BROCKE_APOPTOSIS_REVERSED_BY_IL6	141	0.530479	2.691533	1.00E-10	8.03E-09	5.91E-09
DKK1	GENTILE_UV_HIGH_DOSE_DN	272	0.473464	2.611366	1.00E-10	8.03E-09	5.91E-09
DKK1	MARZEC_IL2_SIGNALING_UP	109	0.536605	2.590208	1.00E-10	8.03E-09	5.91E-09
DKK1	MILI_PSEUDOPODIA_HAPTOTAXIS_UP	465	0.44073	2.571025	1.00E-10	8.03E-09	5.91E-09
DKK1	BILD_HRAS_ONCOGENIC_SIGNATURE	228	0.474107	2.568254	1.00E-10	8.03E-09	5.91E-09
PIK3C2A	MILI_PSEUDOPODIA_HAPTOTAXIS_UP	465	0.606193	3.546977	1.00E-10	7.21E-09	5.87E-09
PIK3C2A	GENTILE_UV_HIGH_DOSE_DN	272	0.484272	2.661034	1.00E-10	7.21E-09	5.87E-09
PIK3C2A	HAMAI_APOPTOSIS_VIA_TRAIL_DN	184	-0.46601	-2.51715	1.00E-10	7.21E-09	5.87E-09
PIK3C2A	CARRILLOREIXACH_MRS3_VS_LOWER_RISK _HEPATOBLASTOMA_DN	146	0.480044	2.409353	1.00E-10	7.21E-09	5.87E-09
PIK3C2A	THUM_SYSTOLIC_HEART_FAILURE_UP	379	0.421499	2.40702	1.00E-10	7.21E-09	5.87E-09
UNC5B	MILI_PSEUDOPODIA_HAPTOTAXIS_UP	465	-0.45431	-2.74541	1.00E-10	1.39E-08	1.11E-08
UNC5B	BROCKE_APOPTOSIS_REVERSED_BY_IL6	141	-0.50696	-2.60194	1.00E-10	1.39E-08	1.11E-08
UNC5B	GENTILE_UV_HIGH_DOSE_DN	272	-0.431	-2.46289	1.00E-10	1.39E-08	1.11E-08
UNC5B	MARZEC_IL2_SIGNALING_UP	109	-0.49338	-2.43836	3.41E-10	4.14E-08	3.31E-08
UNC5B	NEMETH_INFLAMMATORY_RESPONSE_LPS_UP	82	-0.52206	-2.4383	1.71E-09	1.73E-07	1.39E-07
DCN	MILI_PSEUDOPODIA_HAPTOTAXIS_UP	465	0.544796	3.233357	1.00E-10	8.78E-09	7.14E-09
DCN	CARRILLOREIXACH_MRS3_VS_LOWER_RISK _HEPATOBLASTOMA_DN	146	0.496841	2.544813	1.00E-10	8.78E-09	7.14E-09
DCN	HAMAI_APOPTOSIS_VIA_TRAIL_DN	184	-0.44525	-2.47854	1.00E-10	8.78E-09	7.14E-09
DCN	MAHAJAN_RESPONSE_TO_IL1A_DN	65	0.554927	2.444927	8.13E-09	4.43E-07	3.60E-07
DCN	TURASHVILI_BREAST_LOBULAR_CARCINOMA_VS_DUCTAL _NORMAL_UP	66	0.549707	2.442312	1.72E-08	8.45E-07	6.87E-07

GSEA，Gene Set Enrichment Analysis

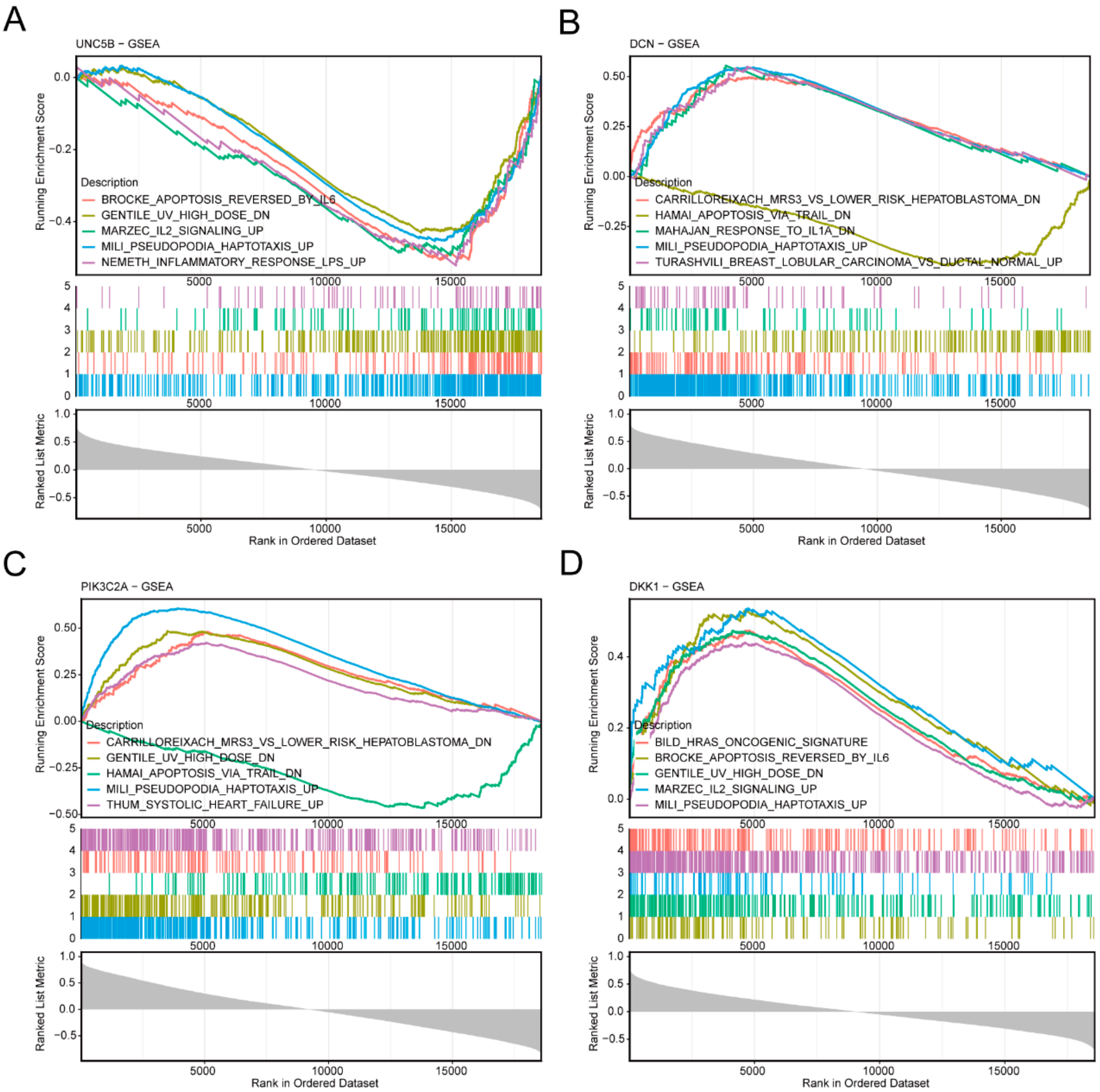

Figure S7. Single-Gene GSEA

A-D. Results of Single-Gene gene set enrichment analysis (single-gene GSEA) of Model Genes *UNC5B* (A), *DCN* (B), *PIK3C2A* (C), and *DKK1* (D) in dataset GSE15197. GSEA, Gene Set Enrichment Analysis.

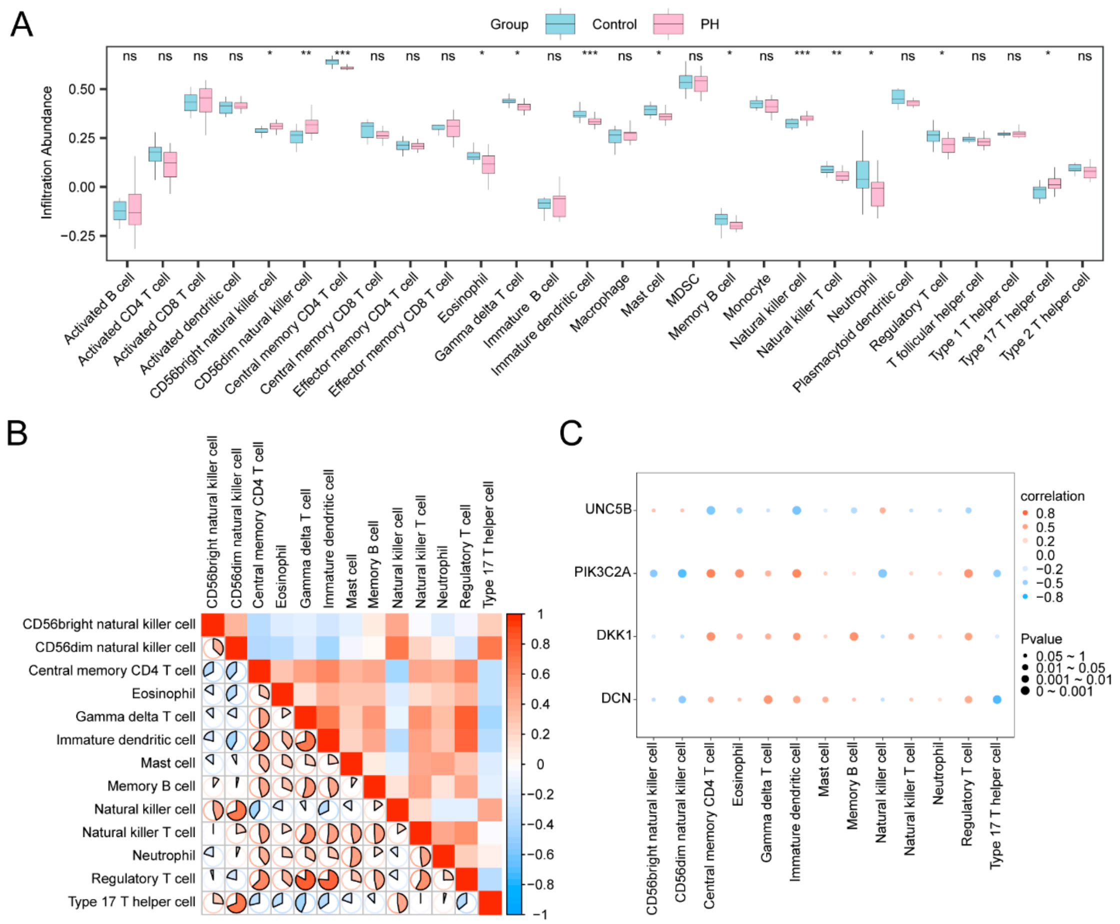

Figure S8. Immune Infiltration Analysis by ssGSEA Algorithm

A. Comparative visualization of immune cell clustering patterns in the dataset GSE15197. B. Heatmap illustrating pairwise correlations among immune cell populations in the same dataset. C. Bubble plot depicting the associations between immune cell infiltration levels and model genes in GSE15197. The notation ns indicates $p \geq 0.05$ (no statistical significance); * indicates $p < 0.05$ (statistical significance); ** indicates $p < 0.01$ (high statistical significance); *** indicates $p < 0.001$ (extremely high statistical significance). Correlation coefficients (r values) are classified as follows: absolute values below 0.3 are considered weak or negligible; values between 0.3 and 0.5 are classified as weak; those between 0.5 and 0.8 are deemed moderate; and those exceeding 0.8 are regarded as strong. In the plots, red indicates positive correlations, while blue indicates negative correlations, with color intensity reflecting the magnitude of the correlation.

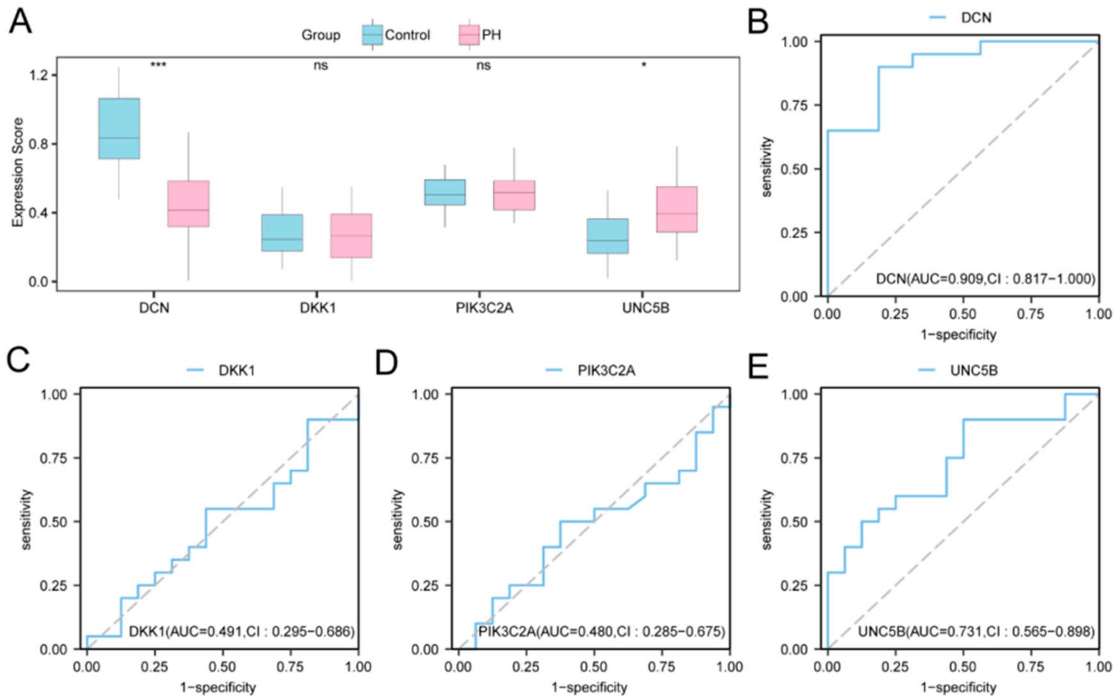

Figure S9. Differential Expression and ROC Curve in dataset GSE1909

A. Group comparison plot of Model Genes in PH and Control of dataset GSE1909. B-E. ROC curves of Model Genes *Dcn* (B), *Dkk1* (C), *Pik3c2a* (D), *Unc5b* (E) in dataset GSE1909. The notation ns indicates $p \geq 0.05$ (no statistical significance); * indicates $p < 0.05$ (statistical significance); ** indicates $p < 0.01$ (high statistical significance); *** indicates $p < 0.001$ (extremely high statistical significance). The diagnostic performance improves as the AUC values approach 1. An AUC value between 0.5 and 0.7 suggests low accuracy, between 0.7 and 0.9 indicates moderate accuracy, and above 0.9 signifies high accuracy. In the group comparison plot, the pink color represents the PH group, while blue corresponds to the Control group.

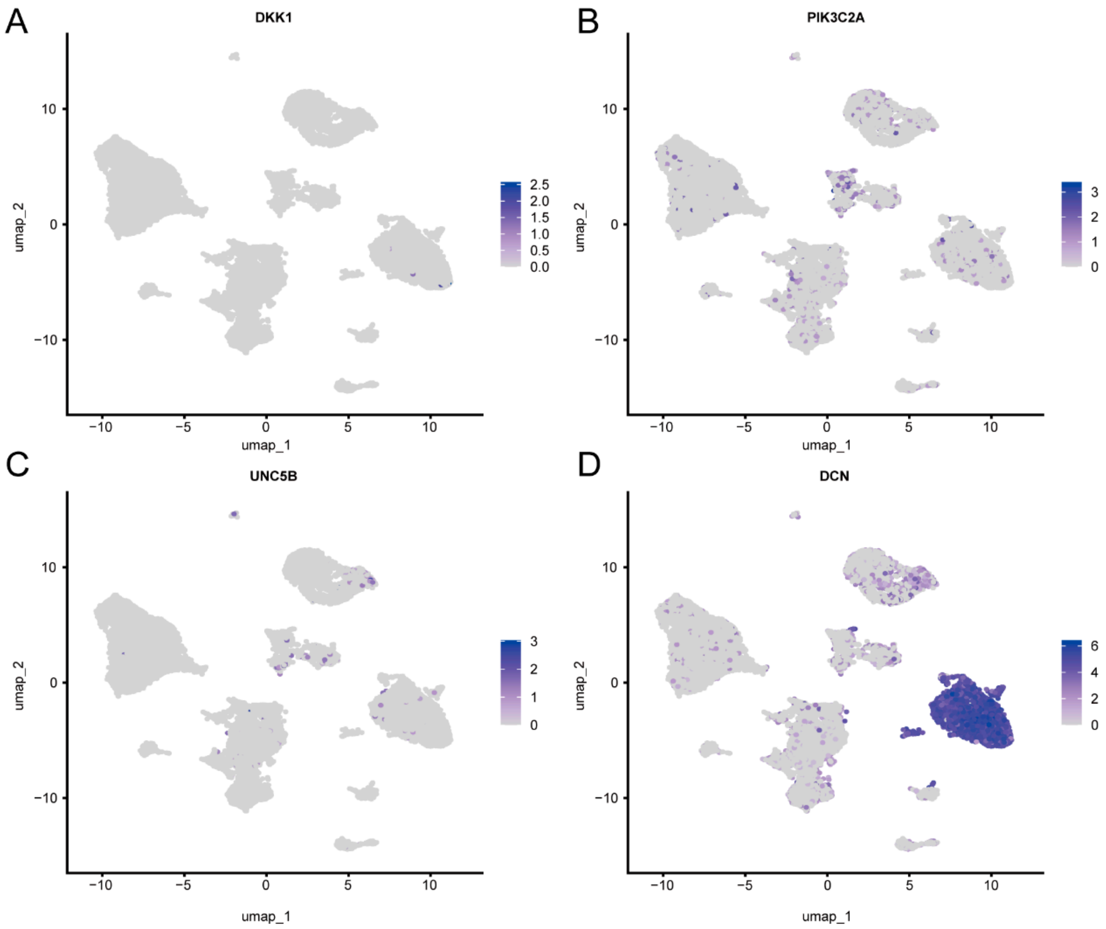

Figure S10. Expression of Model Genes in GSE210248

A-D. UMAP shows the expression levels of Model Genes *DKK1* (A), *PIK3C2A* (B), *UNC5B* (C), and *DCN* (D) in the single cell dataset GSE210248. UMAP, Uniform Manifold Approximation and Projection.

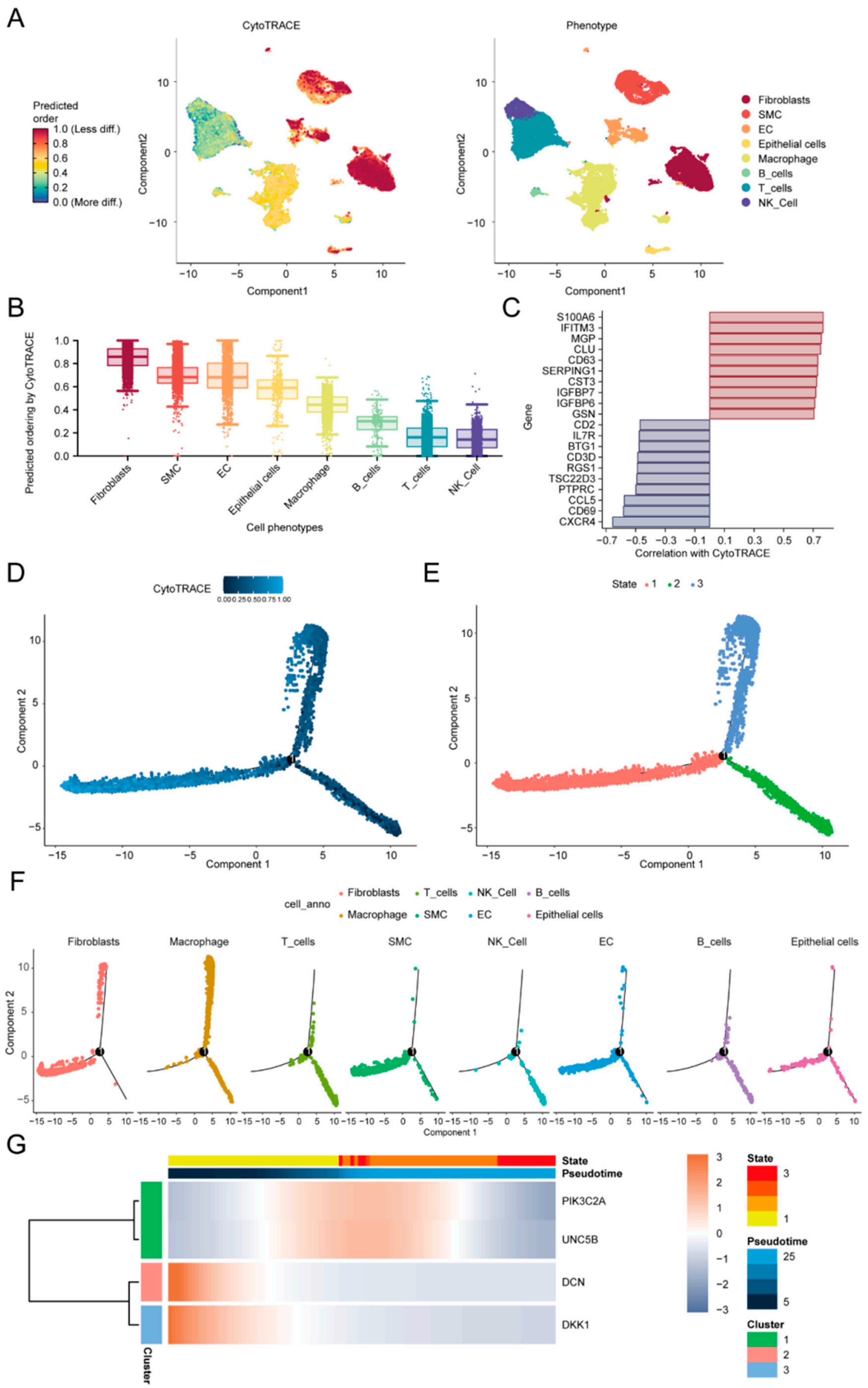

Figure S11. Pseudotime Analysis

A-B. Visual presentation of the UMAP plot (A) and boxplot (B) of CytoTRACE scores between cell populations. The redder the color, the higher the CytoTRACE score, the lower the degree of differentiation, and the stronger the differentiation potential. C. Two-value bar graph of correlation analysis between genes and CytoTRACE score. Red represents positive correlation and purple represents negative correlation. D. Time sequence diagram of cell differentiation and development. Color from dark to light is the order of time. E. Diagram of the differentiation and development trajectory of cells. F. Distribution map of 8 cell types in the cell trajectory skeleton. G. Time-series expression heatmap of Model Genes. In the heat map, orange represents high expression, blue represents low expression, and color depth represents the degree of expression.

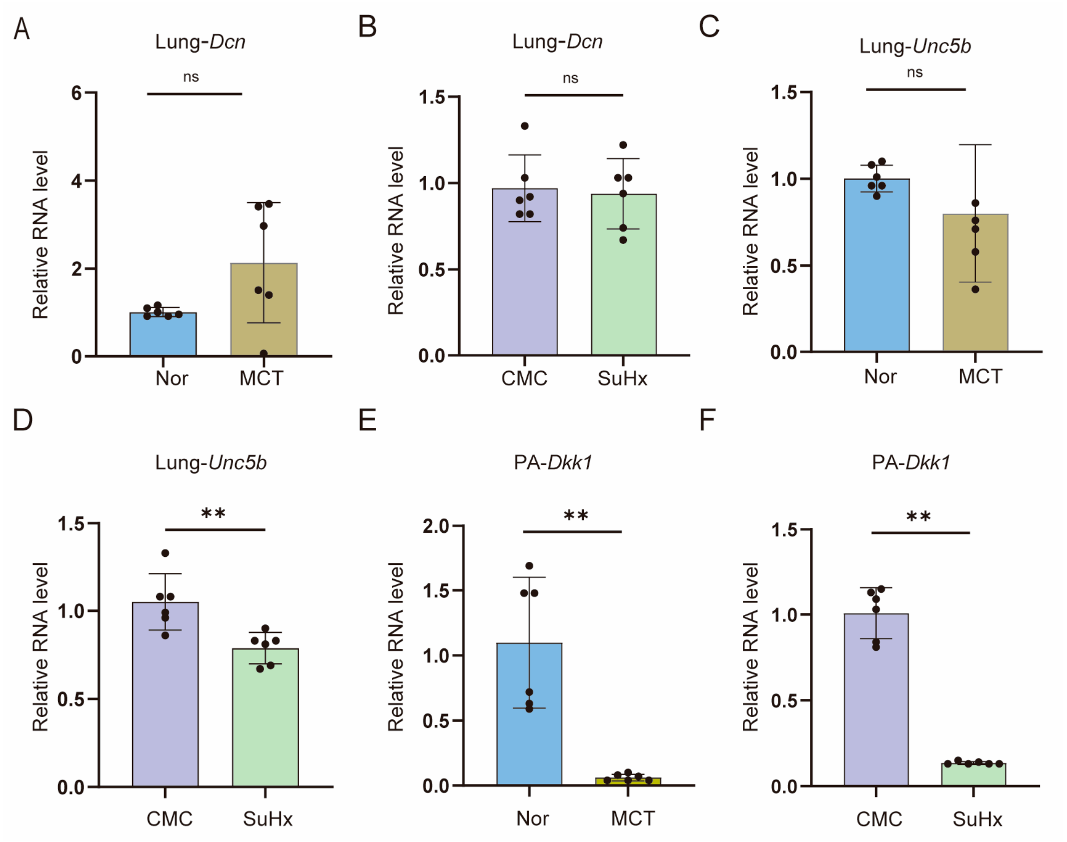

Figure S12. Relative RNA expression levels of *Dcn*, *Unc5b*, and *Dkk1* in rat PH models.

A-B. Expression level of Dcn in the lung tissue of rat PH models validated by qRT-PCR. C-D. Expression level of Unc5b in the lung tissue of rat PH models validated by qRT-PCR. E-F. Expression level of Dkk1 in the pulmonary arteries of rat PH models validated by qRT-PCR. Data are shown as mean ± SEM (n = 6 per group). Nor: normoxia control(for MCT); MCT: monocrotaline-induced model; CMC: carboxymethyl cellulose vehicle control (for SuHx); SuHx: Sugen 5416 + chronic hypoxia model. Relative expression was normalized to β-actin. **P < 0.01,***P < 0.001, ns denotes no statistically significant difference.
