# Supplementary material for: Machine Learning-Based Screening and Experimental Validation of PIK3C2A as a Candidate Angiogenesis-Related Molecule in Pulmonary Hypertension
Source: Int J Mol Sci. 2026 Oct 22;27(21):9421. doi: 10.3390/ijms27219421 (PMC13762966; doi:10.3390/ijms27219421)
Supplement: Supplementary file 1 [file ijms-27-09421-s001.zip › Table S4-GSVA.pdf]

Table S4. GSVA results for high-score versus low-score PH samples

	logFC	AveExpr	t	P.Value	adj.P.Val	B
REACTOME_SARS_COV_2_TARGETS_PDZ_PROTEINS_IN_CELL_CELL_JUNCTION	1.116767	0.059004	7.096538	5.51E-08	0.000179	8.20252
KEGG_MEDICUS_PATHOGEN_HTLV_1_P12_TO_ANTIGEN_PROCESSING_AND_PRESENTATION_BY_MHC_CLASS_I_MOLECULES	0.909824	0.005298	3.733401	0.000758	0.027632	-0.52554
KEGG_MEDICUS_VARIANT_MUTATION_CAUSED_ABERRANT_SNCA_TO_PERK_ATF4_SIGNALING_PATHWAY	0.88845	0.006219	6.902593	9.44E-08	0.000179	7.715533
REACTOME_SYNTHESIS_OF_DIPHTHAMIDE_EEF2	0.826243	0.013902	5.310261	8.70E-06	0.003665	3.578004
KEGG_MEDICUS_VARIANT_MUTATION_CAUSED_ABERRANT_SOD1_TO_PERK_ATF4_SIGNALING_PATHWAY	0.788737	-0.01372	5.321712	8.41E-06	0.003665	3.608293
REACTOME_MASTL_FACILITATES_MITOTIC_PROGRESSION	0.777014	-0.06422	4.975797	2.27E-05	0.004823	2.69327
REACTOME_CYTOSOLIC_TRNA_AMINOACYLATION	0.767762	-0.05207	5.098032	1.60E-05	0.004543	3.016521
WP_DISORDERS_IN_KETONE_BODY_SYNTHESIS	0.764051	-0.06243	5.391313	6.89E-06	0.003665	3.792338
WP_KETONE_BODIES_SYNTHESIS_AND_DEGRADATION	0.764051	-0.06243	5.391313	6.89E-06	0.003665	3.792338
REACTOME_PROPIONYL_COA_CATABOLISM	0.753084	-0.08838	3.813396	0.000608	0.026521	-0.32515

KEGG_MEDICUS_REFERENCE_TRANSPORT_OF_CALCIUM	-0.64983	0.025465	-3.78764	0.000653	0.02692	-0.38982
REACTOME_LECTIN_PATHWAY_OF_COMPLEMENT_ACTIVATION	-0.6724	-0.00845	-4.37885	0.000125	0.01448	1.124535
KEGG_MEDICUS_REFERENCE_LECTIN_PATHWAY_OF_COAGULATION_CASCADE_PROTHROMBIN_TO_THROMBIN	-0.68243	0.001566	-4.62568	6.20E-05	0.010687	1.770275
BIOCARTA_PPARG_PATHWAY	-0.68645	-0.03112	-4.27805	0.000166	0.015025	0.862634
REACTOME_THE_FATTY_ACID_CYCLING_MODEL	-0.70944	0.073494	-3.89946	0.00048	0.024105	-0.10803
BIOCARTA_DICER_PATHWAY	-0.75287	0.06345	-2.80922	0.008502	0.092076	-2.70513
REACTOME_POST_TRANSCRIPTIONAL_SILENCING_BY_SMALL_RNAS	-0.79663	0.111189	-3.43072	0.001717	0.041219	-1.26954
KEGG_MEDICUS_REFERENCE_MANNOSE_TYPE_O_GLYCAN_BIOSYNTHESIS_POMT_TO_POMK	-0.80075	0.029588	-3.62073	0.00103	0.032284	-0.80526
REACTOME_FICOLINS_BIND_TO_REPETITIVE_CARBOHYDRATE_STRUCTURES_ON_THE_TARGET_CELL_SURFACE	-0.83243	-0.00334	-5.18931	1.23E-05	0.004245	3.25802
BIOCARTA_PELP1_PATHWAY	-0.85656	-0.03841	-5.25369	1.02E-05	0.003881	3.428356

GSVA，Gene Set Variation Analysis。
