# Supplementary material for: Machine Learning-Based Screening and Experimental Validation of PIK3C2A as a Candidate Angiogenesis-Related Molecule in Pulmonary Hypertension
Source: Int J Mol Sci. 2026 Oct 22;27(21):9421. doi: 10.3390/ijms27219421 (PMC13762966; doi:10.3390/ijms27219421)
Supplement: Supplementary file 1 [file ijms-27-09421-s001.zip › Table S5-GSEA.pdf]

Table S5. Single-gene GSEA results for model genes

Gene	ID	setSize	enrichmentScore	NES	pvalue	p.adjust	qvalue
DKK1	BROCKE_APOPTOSIS_REVERSED_BY_IL6	141	0.530479	2.691533	1.00E-10	8.03E-09	5.91E-09
DKK1	GENTILE_UV_HIGH_DOSE_DN	272	0.473464	2.611366	1.00E-10	8.03E-09	5.91E-09
DKK1	MARZEC_IL2_SIGNALING_UP	109	0.536605	2.590208	1.00E-10	8.03E-09	5.91E-09
DKK1	MILI_PSEUDOPODIA_HAPTOTAXIS_UP	465	0.44073	2.571025	1.00E-10	8.03E-09	5.91E-09
DKK1	BILD_HRAS_ONCOGENIC_SIGNATURE	228	0.474107	2.568254	1.00E-10	8.03E-09	5.91E-09
PIK3C2A	MILI_PSEUDOPODIA_HAPTOTAXIS_UP	465	0.606193	3.546977	1.00E-10	7.21E-09	5.87E-09
PIK3C2A	GENTILE_UV_HIGH_DOSE_DN	272	0.484272	2.661034	1.00E-10	7.21E-09	5.87E-09
PIK3C2A	HAMAI_APOPTOSIS_VIA_TRAIL_DN	184	-0.46601	-2.51715	1.00E-10	7.21E-09	5.87E-09
PIK3C2A	CARRILLOREIXACH_MRS3_VS_LOWER_RISK _HEPATOBLASTOMA_DN	146	0.480044	2.409353	1.00E-10	7.21E-09	5.87E-09
PIK3C2A	THUM_SYSTOLIC_HEART_FAILURE_UP	379	0.421499	2.40702	1.00E-10	7.21E-09	5.87E-09
UNC5B	MILI_PSEUDOPODIA_HAPTOTAXIS_UP	465	-0.45431	-2.74541	1.00E-10	1.39E-08	1.11E-08
UNC5B	BROCKE_APOPTOSIS_REVERSED_BY_IL6	141	-0.50696	-2.60194	1.00E-10	1.39E-08	1.11E-08
UNC5B	GENTILE_UV_HIGH_DOSE_DN	272	-0.431	-2.46289	1.00E-10	1.39E-08	1.11E-08

UNC5B	MARZEC_IL2_SIGNALING_UP	109	-0.49338	-2.43836	3.41E-10	4.14E-08	3.31E-08
UNC5B	NEMETH_INFLAMMATORY_RESPONSE_LPS_UP	82	-0.52206	-2.4383	1.71E-09	1.73E-07	1.39E-07
DCN	MILI_PSEUDOPODIA_HAPTOTAXIS_UP	465	0.544796	3.233357	1.00E-10	8.78E-09	7.14E-09
DCN	CARRILLOREIXACH_MRS3_VS_LOWER_RISK _HEPATOBLASTOMA_DN	146	0.496841	2.544813	1.00E-10	8.78E-09	7.14E-09
DCN	HAMAI_APOPTOSIS_VIA_TRAIL_DN	184	-0.44525	-2.47854	1.00E-10	8.78E-09	7.14E-09
DCN	MAHAJAN_RESPONSE_TO_IL1A_DN	65	0.554927	2.444927	8.13E-09	4.43E-07	3.60E-07
DCN	TURASHVILI_BREAST_LOBULAR_CARCINOMA_VS_DUCTAL _NORMAL_UP	66	0.549707	2.442312	1.72E-08	8.45E-07	6.87E-07

GSEA，Gene Set Enrichment Analysis。
